# Stereoisomer library prepared *via* controlled radical polymerization: isolation, structural identification and discovery of stereospecific gelation behaviour of tri(N-phenyl acrylamide)†

**DOI:** 10.1039/d5sc00612k

**Published:** 2025-05-20

**Authors:** Yukiko Nagai, Hinako Iwamoto, Satoki Fukuda, Sotaro Akashi, Shota Iseri, Hayato Tada, Tomohiro Yamanaka, Konosuke Wada, Sotaro Tsuji, Toshikazu Ono, Yoshiko Miura, Tohru Taniguchi, Yu Hoshino

**Affiliations:** a Department of Applied Chemistry, Graduate School of Engineering, Kyushu University 744 Motooka Nishi-ku Fukuoka 819-0395 Japan hoshino.yu.673@m.kyushu-u.ac.jp; b Department of Chemical Engineering, Graduate School of Engineering, Kyushu University 744 Motooka Nishi-ku Fukuoka 819-0395 Japan; c Department of Advanced Transdisciplinary Sciences, Faculty of Advanced Life Science, Hokkaido University Kita 10, Nishi 8, Kita-ku Sapporo 060-0810 Japan; d Center for Molecular Systems (CMS), Kyushu University 744 Motooka Nishi-ku Fukuoka 819-0395 Japan

## Abstract

The construction of stereoisomer libraries is essential for designing, maximizing, and tuning the biological, self-assembly, and photoelectromagnetic properties of optically active molecules. In this study, a stereoisomer library consisting of all eight stereoisomers of a vinyl oligomer, a trimer of phenyl acrylamide (tri(PAAm)), was prepared by a one-pot controlled radical polymerization reaction followed by two-step column chromatography. Tacticity of each isomer, *i.e.*, the relative stereochemistry of three chiral centers within tri(PAAm), was assigned using proton nuclear magnetic resonance (^1^H-NMR) and circular dichroism (CD) spectra. Interestingly, one stereoisomer self-assembled and formed an organo-gel. The control experiments revealed that the assembly was driven *via* configuration-dependent intermolecular hydrogen bonding and π–π interaction. The absolute structure of the gelled tri(PAAm) was identified by combining the tacticity data with the CD spectra and an electronic CD spectrum calculated by density functional theory (DFT). Stereoisomer libraries easily prepared *via* one-pot radical polymerization are promising modalities for supramolecular chemistry, pharmaceuticals, and photoelectromagnetic materials.

## Abstract 2

The construction of stereoisomer libraries is essential for designing the properties of optically active molecules. In this study, a stereoisomer library was prepared by a controlled radical polymerization reaction followed by column chromatography.

## Graphical abstract

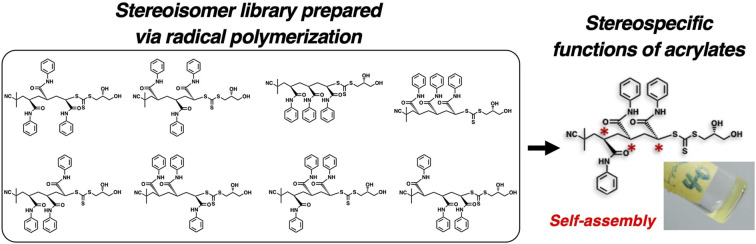

## Introduction

The construction of stereoisomer libraries is essential for designing, maximizing, and tuning properties of biologically and photo-electromagnetically active compounds. Dennis P. Curran *et al.* have developed a method for preparing stereoisomer libraries, which they called fluorous mixture synthesis (FMS),^1^ by combining fluorine labelling of compounds with fluorous silica gel chromatography.^2–5^ K. Hayashi *et al.* constructed a stereoisomer library of plant hormone analogs through multistep reaction and selected agonists with affinity for receptor proteins.^6^ Dave J. Adams prepared a stereoisomer library of a dipeptide through 4 step reaction and purification processes and discovered that the peptides self-assembled into different supramolecular structures depending on the stereochemistry of the peptides.^7^ Despite the importance of stereoisomer libraries, constructing stereoisomer libraries for compounds with multiple chiral centers still requires multi-step reactions, labelling, protection, deprotection, and purification processes.

Controlled radical polymerization enables the formation of numerous C–C bonds with a multiple chiral center in a single reaction. In recent years, the synthesis of oligomers with highly narrow molecular weight distributions has been achieved by combining chain-growth vinyl radical addition reactions with reversible chain transfer and atom transfer reactions.^8^ Subsequent separation by chromatography isolated oligomers with precise molecular weights and monomer sequences.^9,10^ Due to discrete molecular weight, the isolated “precision” oligomers exhibit distinct optical, thermal, and physicochemical properties.^11–14^ Furthermore, precision oligomers with specific affinities to target biomolecules were identified by affinity screening of the precision block-oligomer libraries.^15,16^

Despite advances in precise/discrete oligomer libraries, constructing oligomer libraries with defined stereochemistry remains elusive. Polymerization of representative monomers, such as acrylic monomers, introduces asymmetric carbons into the main chain depending on the number of monomer units in the resulting polymers. The number of stereoisomers created *via* a single polymerization reaction is 2^*n*^ when the number of monomer units in a polymer is *n*. Although the synthesis of highly stereo-regular polymers^17–20^ and precision oligomers^21^ has been reported, a stereoisomer library of precise oligomers has not been constructed.

**Scheme 1:**
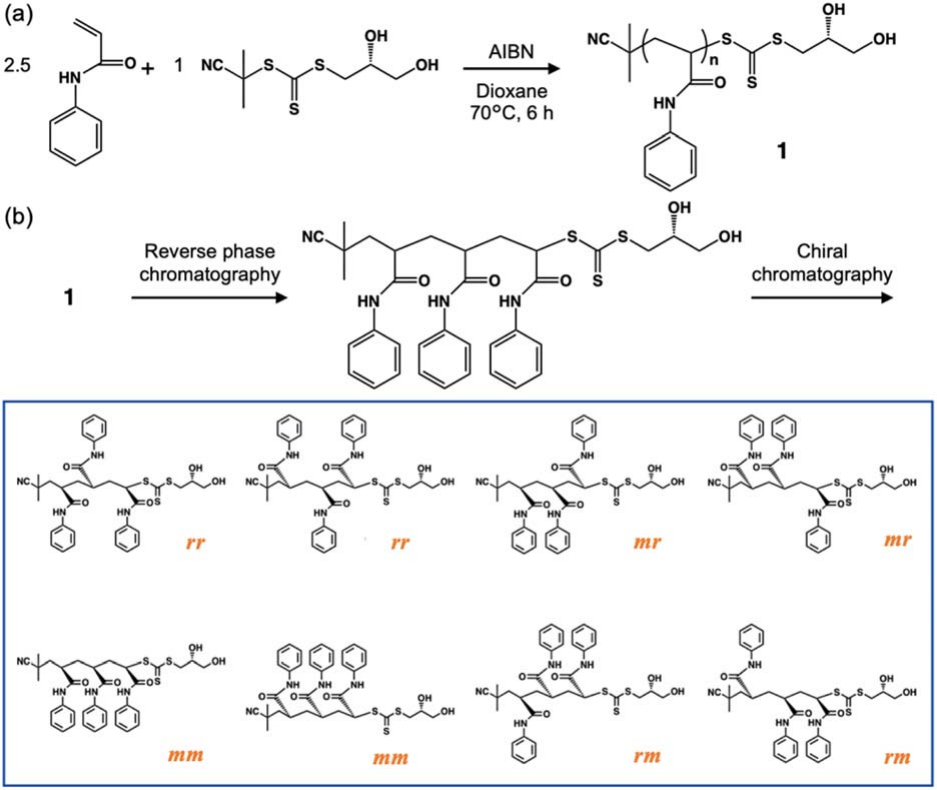
Preparation of stereoisomer library *via* radical polymerization. (a) Synthesis of oligo(*N*-phenyl acrylamide) with narrow molecular weight distribution. (b) Preparation of tri(PAAm) stereoisomer library *via* molecular weight-based separation by reverse phase chromatography and stereochemistry-based separation by chiral chromatography of the oligo(*N*-phenyl acrylamide). The *m* and *r* stand for *meso* and *rasemo* diad, respectively.

In this study, we report the facile preparation of the stereoisomer library with three chiral centers *via* controlled radical polymerization followed by two-step column chromatography. Specifically, *N*-phenyl acrylamide oligomers with a narrow molecular weight distribution were synthesized by one-pot reversible addition–fragmentation chain transfer (RAFT) polymerization, and all eight stereoisomers were isolated on ∼10 mg scale by a combination of reverse phase and chiral chromatography (Scheme 1).

The stereochemistry of the eight stereoisomers was identified through ^1^H NMR, electrospray ionization mass spectra (ESI-MS), CD spectra, and DFT calculations. Interestingly, only one stereoisomer among the eight stereoisomers exhibited self-assembly in a specific solvent environment, causing a gelation of the solution. This study presents the first report on preparing a stereoisomer library of vinyl oligomers and its stereochemistry-dependent gelation behavior.

## Results and discussion

The process of preparing the PAAm stereoisomer library is depicted in Scheme 1. PAAm was selected as a model compound because the rigid planar sidechain of the phenylamide^22^ allows the properties of the PAAm oligomer to reflect the chirality of the main chain. In addition, PAAm was selected because it absorbs the UV-visible light, making it possible to identify the stereoisomer configurations by CD spectroscopy. PAAm was polymerized at 70 °C for 6 hours in the presence of (R)-2-cyanopropan-2-yl (2,3-dihydroxypropyl) carbonotrithioate ((R)-CPDOTT)) (see ESI, Fig. S1–S6†) and azobis(isobutyronitrile) (AIBN) as a chain transfer agent (CTA) and free radical initiator, respectively, for RAFT polymerization. CPDOTT contains hydroxyl groups in its end group, enabling the isolation of discrete oligomers that contained hydrophobic groups on their side chains by low-pressure reverse-phase chromatography.^11,17^ An asymmetric carbon to which the 2-hydroxy group is attached was introduced in the CTA to assist the separation of stereoisomers by converting the enantiomers of the oligomer into diastereomers. Furthermore, the structure of the radical species from CPDOTT in the first chain-transfer reaction was designed to be the same as the initiator radicals derived from AIBN, avoiding the formation of oligomers with non-uniform end groups. The stoichiometry between PAAm and CTA was adjusted to be 2.5 so that the dimer and trimer of PAAm would be synthesized as a major product. The monomer conversion was determined to be 100% by ^1^H NMR (Fig. S7†).

**Fig. 1:**
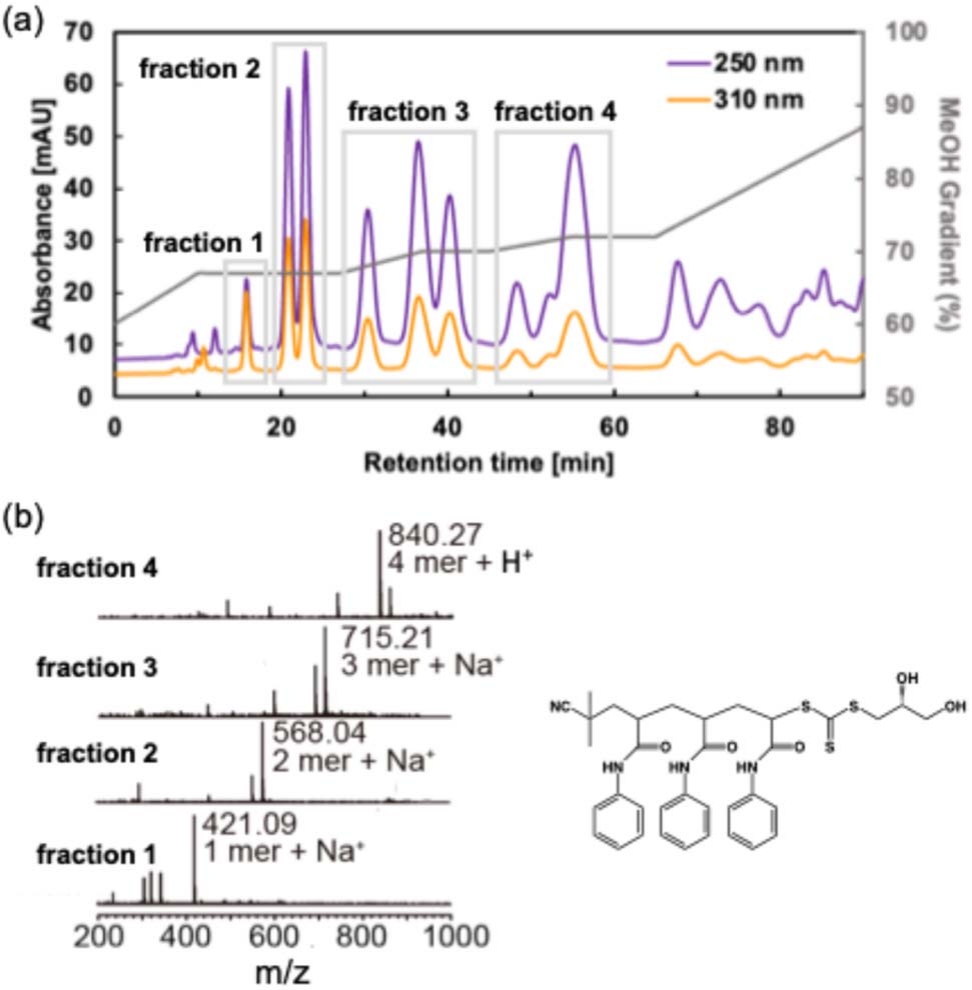
Isolation of tri(PAAm). (a) Fractionation of oligo(*N*-phenyl acrylamide) by reverse phase chromatography. The chromatogram was monitored by the absorption of the phenyl group (250 nm, purple line) and trithiocarbonate group (310 nm, orange line). Water (100-X%) and methanol (X%) were used as the mobile phase. The volume fraction of methanol (X%) is shown in a gray line. (b) ESI†-MS spectra of the fractions 1 to 4. Insert: chemical structure of tri(PAAm).

**Fig. 2:**
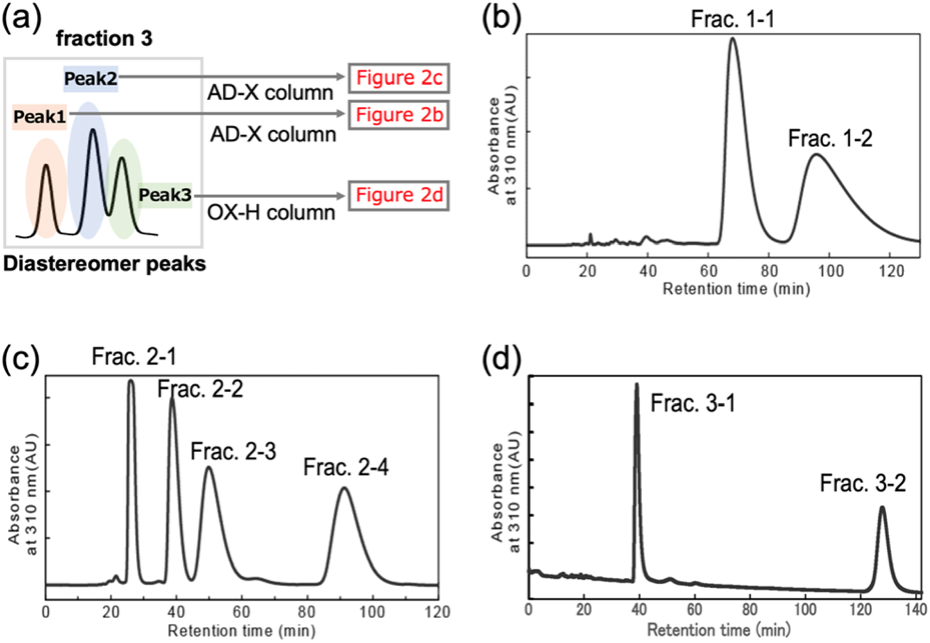
Isolation of stereoisomers of the tri(PAAm). (a) Schematic illustration of the experimental outlines. Peak 1, 2, and 3 of tri(PAAm) isolated *via* reverse phase chromatography were further separated *via* normal phase chiral chromatography. Normal phase chiral chromatogram of the tri(PAAm) diastereomer (b) peak 1, (c) peak 2, and (d) peak 3.

PAAm oligomers synthesized by RAFT polymerization were first separated using reversed-phase high-performance liquid chromatography (HPLC) based on their affinity for octadecyl silyl (ODS) silica gel (Fig. 1a). The chromatogram was monitored by the absorption of the phenyl group (absorption wavelength: 250 nm, purple line in Fig. 1a) and trithiocarbonate group (absorption wavelength: 310 nm, orange line in Fig. 1a). An ODS silica gel column (diameter: 50 mm, length: 250 mm) was used to separate 750 mg of the oligomers. Here, water and methanol were used as the mobile phase. The peaks in the chromatogram were fractionated, and the molecular weight of the oligomer in each fraction was identified by ESI†-MS (Fig. 1b). The molar mass of the solute in a peak at 16 min (fraction 1) was 421, which matched the single PAAm adducts ionized with Na^+^. The mass of the next two peaks eluted between 20 and 25 min (fraction 2) was 568, which matched the two PAAm adducts ionized with Na^+^. The mass in the following three peaks eluted between 27 and 42 min (fraction 3) was 715, which matched the three PAAm adducts (tri(PAAm)) ionized with Na^+^. That of the following three peaks eluted between 45 and 60 min (fraction 4) was 804, which matched the four PAAm adducts ionized with H^+^. To construct a stereoisomer library of tri(PAAm), three peaks in fraction 3, denoted as peak 1, peak 2, and peak 3, were isolated (Fig. 2a). The yields were 20 mg, 27 mg, and 13 mg, respectively. ^1^H NMR, ESI†-MS, and UV-vis spectra indicate the three peaks include diastereomer of tri(PAAm) (Fig. S8 and S9†).

The three diastereomer peaks of tri(PAAm) were then separated based on differences in stereochemistry using normal-phase chiral HPLC. As shown in Fig. 2a, peak 1 (orange) and peak 2 (blue) were separated by CHIRAL PAK AD-H column (diameter: 30 mm, length: 250 mm) with hexane/ethanol = 70/30, and peak 3 (green) was separated by CHIRALCEL OX-H column (diameter: 30 mm, length: 250 mm) with acetonitrile as an elution solvent. Two peaks were fractionated with almost the same amount, 8.2 mg and 8.1 mg for Frac. 1–1 and Frac. 1–2, respectively, when the peak 1 was separated by chiral HPLC (Fig. 2b). When the peak 2 was separated by the chiral HPLC, four peaks were fractionated with almost the same amount, 4.2 mg, 4.4 mg, 4.1 mg, and 3.9 mg for Frac. 2–1, Frac. 2–2, Frac. 2–3, and Frac. 2–4, respectively. The peak 3 was separated into two fractions; 4.1 mg and 4.4 mg for Frac. 3–1 and Frac. 3–2, respectively. ESI†-MS confirmed the molar mass of the oligomers to be 715 in all eight fractions, which matched the tri(PAAm) ionized with Na^+^ (Fig. S13–20†).

**Fig. 3:**
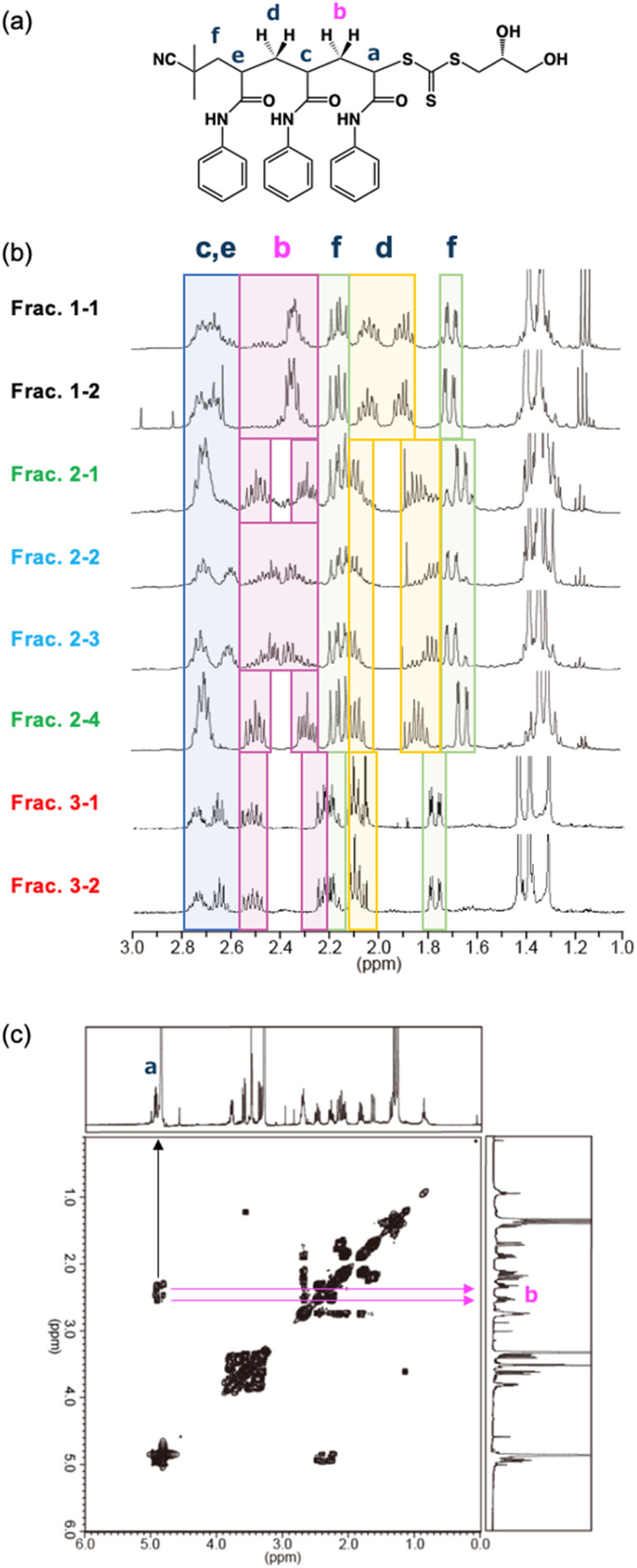
(a) Structural formula of tri(PAAm). (b) ^1^H NMR spectra of eight fractions of tri(PAAm) in the stereoisomer library. Full-scale ^1^H NMR spectra were shown in Fig. S13–20†. (c) ^1^H-^1^H COSY spectrum of tri(PAAm) in Frac. 2–1. The assigned stereochemistry for all fractions were shown in Fig. S22.†

The relative configuration of adjacent chiral centers of all eight stereoisomers of tri(PAAm) was determined from ^1^H NMR spectra and ^1^H-^1^H correlation spectroscopy (COSY) of the methine and methylene protons in the main chain of the tri(PAAm) (Fig. 3 and S13–21†). Here, the methine and methylene protons are denoted *H*_a_–*H*_e_ (Fig. 3a). In Frac. 2–1, for example, the quartet around 4.9 pp are coupled with the 2 protons around 2.4 ppm in the ^1^H-^1^H COSY, and observed at a lower magnetic field than the other protons in the main chain (Fig. 3b, 3c and S15†). Therefore, the quartet around 4.9 ppm was assigned to the *H*_a_ next to the sulfur atom of trithiocarbonate. The multiplet around 2.7 ppm was assigned to *H*_c_ and *H*_e_ next to the carbonyl carbon (highlighted by the blue box in Fig. 3b), which has four coupling protons. The septets around 2.5 ppm and 2.3 ppm were assigned *H*_b1_ and *H*_b2_ (highlighted by the magenta boxes in Fig. 3b), respectively, according to the coupling with *H*_a_ in ^1^H-^1^H COSY. The multi-lines around 2.1 ppm included quartet and quintet. The quartet in a lower magnetic field is coupled with a quartet around 1.6 ppm, and the quintet in a higher magnetic field is coupled with a quintet around 1.8 ppm in the ^1^H-^1^H COSY. Thus, we assigned the quantets are *H*_f1_ and *H*_f2_ (highlighted by the green boxes in Fig. 3b), and the quintets are *H*_d1_ and *H*_d2_ (highlighted by the yellow boxes in Fig. 3b), respectively. The different chemical shifts of the two methylene protons of *H*_b_ was derived from meso diad (*m*) of two adjacent side chains. The two protons of *H*_d_ also showed different chemical shifts due to the meso diad (*m*) of side chains. From those observations, we assigned the relative stereochemistry of Frac. 2–1 to be isotactic (*mm*).

Relative stereochemistry (triad) of eight stereoisomers of tri(PAAm) was assigned using the same method. Determining the diad from AIBN terminus to diol terminus; raceme–raceme (*rr*) in Frac. 1–1 and Frac. 1–2, meso–meso (*mm*) in Frac. 2–1 and Frac. 2–4, meso-raceme (*mr*) in Frac. 2–2 and Frac. 2–3, raceme-meso (*rm*) in Frac. 3–1 and Frac. 3–2 were assigned (Fig. S13−22†). According to the ^1^H NMR signals of the methine or methylene protons in the main chain observed in 1–3 ppm, Frac. 1–1 and Frac. 1–2, Frac. 2–1 and Frac. 2–4, Frac. 2–2 and Frac. 2–3, Frac. 3–1 and Frac. 3–2 were shown the same chemical shift, respectively, which indicated that each pair had an enantiomeric relationship in the asymmetric carbons on the tri(PAAm) structure (*C*_a_, *C*_c_, and *C*_e_) (Fig. 3b).

**Fig. 4:**
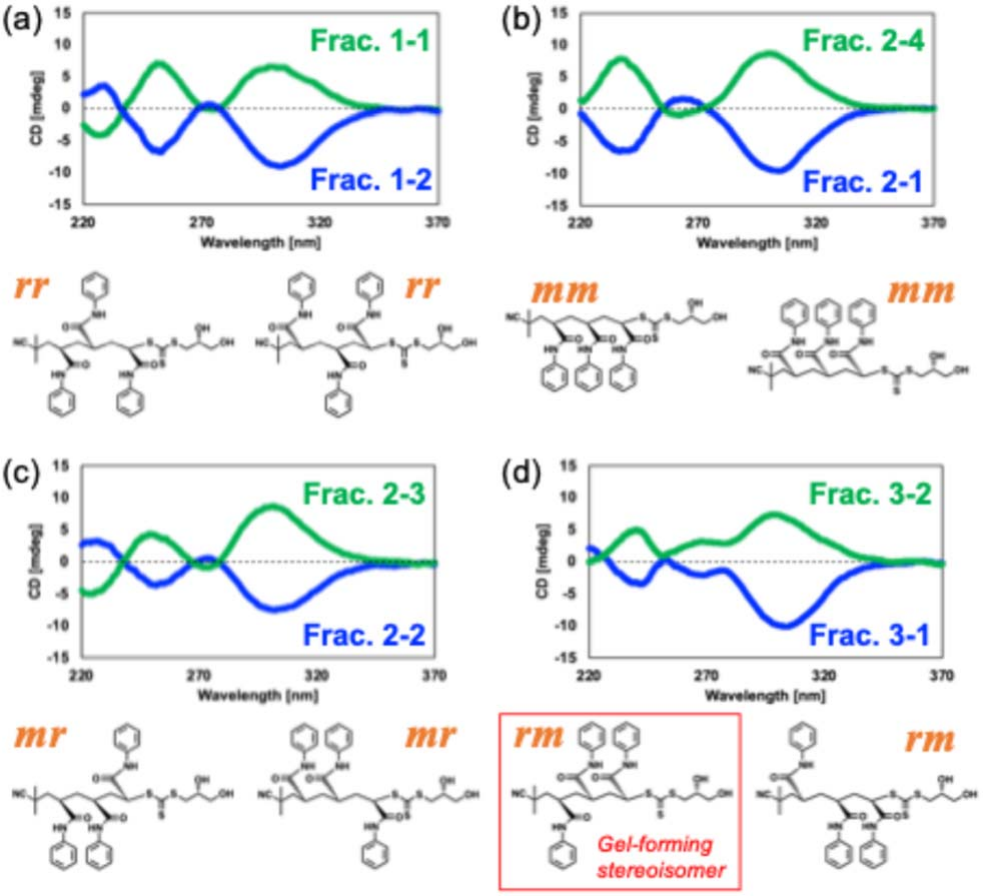
CD spectra for eight fractions of stereoregular tri(PAAm). (a) Frac. 1–1 (green line) and Frac. 1–2 (blue line), (b) Frac. 2–1 (blue line) and Frac. 2–4 (green line), (c) Frac. 2–2 (blue line) and Frac. 2–3 (green line), (d) Frac. 3–1 (blue line) and Frac. 3–2 (green line). Insert: chemical structures of each isomer. Triads are shown in orange.

CD spectra of the isomers were obtained to validate the stereochemistry of these fractions. In CD spectra of each fraction (in methanol absorbance at 0.5, Fig. 4), all fractions showed characteristic peaks at around 250 nm and 310 nm derived from the cotton effect in the main chain. The pairs of opposite CD spectra strongly suggested an enantiomeric relationship in the asymmetric carbons on the tri(PAAm) structure (*C*_a_, *C*_c_, and *C*_e_). CD signals showed linear concentration dependency (Fig. S23 and 24†), indicating that the CD signals reflect the stereochemistry of each isomer in the solution. These results concluded the eight isomers were successfully isolated.

**Fig. 5:**
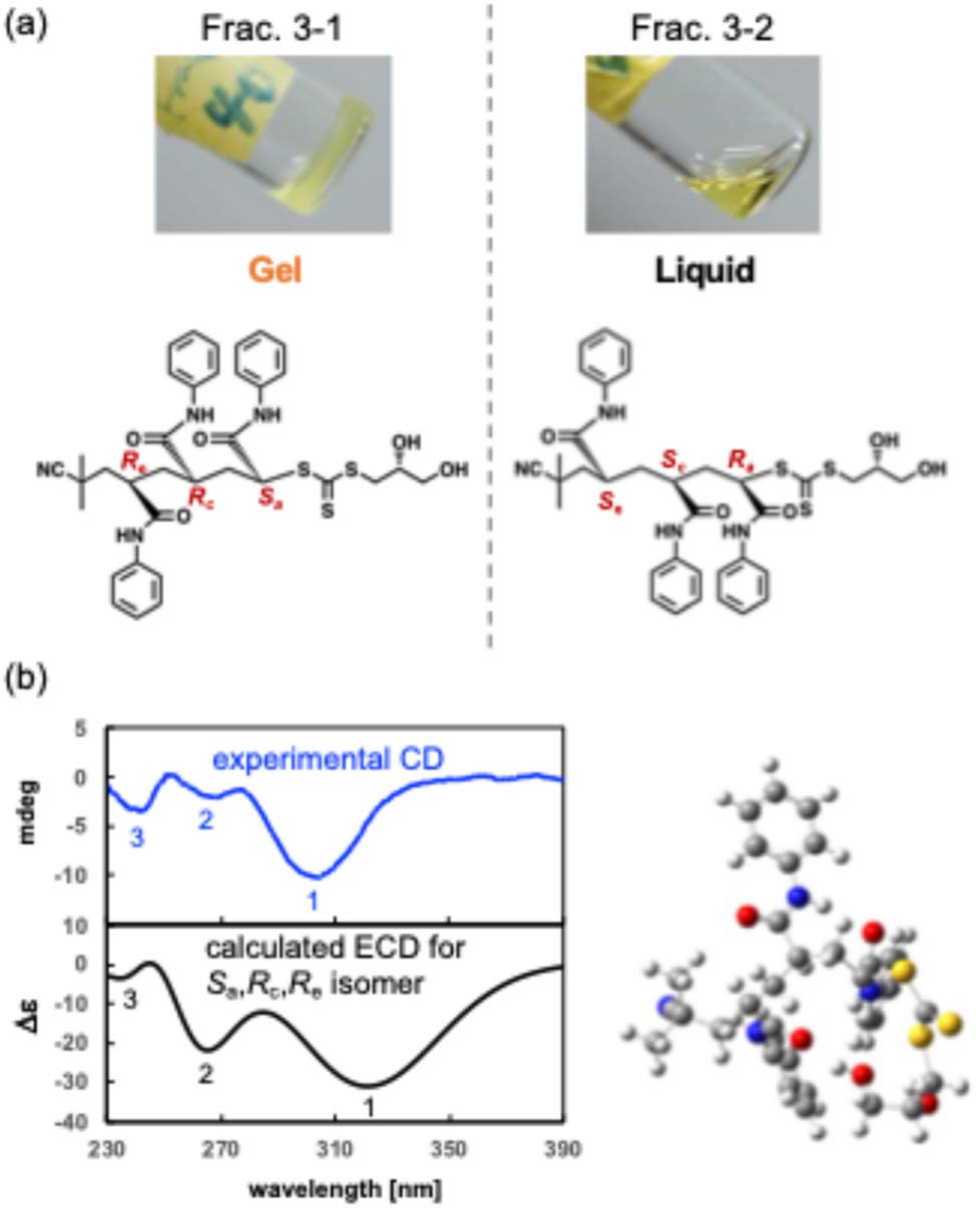
(a) Photographs for the gelation behavior of Frac. 3–1 and Frac. 3–2 in chloroform. Insert chemical structure is tri(PAAm) with absolute configuration “*R*_a_, *S*_c_, *S*_e_”. (b) Elucidation of the absolute configuration of Frac. 3–1 by comparison of the calculated ECD spectrum (black line) with the experimentally measured CD spectrum (blue line).

During detailed characterization of the stereoisomers, a stereochemistry-dependent gelation of tri(PAAm) was discovered. Frac. 3–1 (40 mg mL^−1^) formed a gel in chloroform at 4 °C (Fig. 5a and 4d). In contrast, the other stereoisomers (Fig. 4a–d) showed no gelation in a comparable concentration range (Table S1, Fig. S22†). The gelation behaviors indicate that the stereoisomer in Frac. 3–1 self-assembles into the fiber-like structure and forms a network in the chloroform. Interestingly, other isomers, including the one in Frac. 3–2 did not form gels in the same condition (Fig. 5a), although Frac. 3–1 and Frac 3–2 have the same relative stereochemistry (a triad of *rm*). This result indicates that the stereochemistry of all four asymmetric carbons, including the carbon to which the 2-hydroxy group is attached, governs the self-assembly and gelation of tri(PAAm).

Morphology of assembled tri(PAAm) of Frac. 3–1 in the gel (200 μM in chloroform) was observed by atomic force microscopy (AFM) on a mica (Fig. S25†).^23^ AFM image showed fiber- and network-like structures. The heights of the fibers were 6–8 nm, indicating that the network structure was formed by assembly of 3–4 molecules of tri(PAAm).

The gelation test was conducted in the different solvents to investigate the mechanism of the stereochemistry-dependent gelation behavior of Frac. 3–1. When the solvent with a lower dielectric constant (DI) than chloroform (DI; 4.8), such as hexane (DI; 1.9) and diethyl ether (DI; 4.3), was used instead of chloroform, tri(PAAm) precipitated and did not form gels (Table S3†). On the other hand, when the solvent with higher dielectric constant than chloroform, such as ethyl acetate, tetrahydrofuran (THF), *N*-methyl pyrrolidone (NMP), ethanol, methanol and *N*,*N*-dimethylformamide (DMF) was used, tri(PAAm) dissolved but did not form gels (Table S3†). Those results indicate that the DI has to be high enough to dissolve tri(PAAm) and low enough not to prevent intra-molecular stereochemistry-dependent interaction. Considering that the stereochemistry of the 2-hydroxy group controls gelation (Fig. 5a), the hydrogen bonds of the hydroxy group may be involved in gelation. A small amount (10 vol%) of benzene interfered with the gelation of the tri(PAAm) in chloroform and dissolved the oligomers, although the DI of benzene is low (DI; 2.3). This result indicates that π–π interactions between oligomers were also crucial for gelation.

Finally, the absolute structure of the gelled tri(PAAm) (*rm*) in Frac. 3–1 was identified by combining the tacticity data with the CD spectra and an electronic CD spectrum calculated by DFT. As discussed above, ^1^H NMR analysis indicated its stereochemistry to be racemo-meso, either *R*_a_,*S*_c_,*S*_e_ or *S*_a_,*R*_c_,*R*_e_. Fig. 5b shows the calculated electronic CD spectrum of the *S*_a_,*R*_c_,*R*_e_ stereoisomer (Fig. S26†). The theoretical CD spectrum mostly reproduced the experimentally measured one; a strong negative broad cotton effect at around 310 nm (1 in Fig. 5b) and relatively weak negative signals at around 270 nm and 240 nm (2 and 3 in Fig. 5b), which led the absolute configuration of Frac. 3–1 to be *S*_a_,*R*_c_,*R*_e_.

A further understanding of the precision oligomer self-assembly will provide insights into the rational design of the monomer sequence, molecular weight, and stereochemistry, enabling the modulation of the 1D, 2D and 3D structure of precision oligomers. We propose using the stereoisomer libraries to design, maximize, and tune the properties of biologically and photo-electromagnetically active acrylic polymers.

## Conclusions

In summary, we constructed the stereoisomer library of tri(PAAm) *via* RAFT polymerization followed by reverse phase- and chiral chromatography. The relative stereochemistry of all stereoisomers was assigned by ^1^H NMR and CD spectra. Interestingly, one stereoisomer formed a gel in chloroform at 4 °C. The gelation was not observed in protonic solvents and aprotic solvents with higher dielectric constant than chloroform, probably because the solvents act as hydrogen bond acceptors. Furthermore, benzene inhibited the gelation by interfering with the π–π interaction of phenyl groups. Accordingly, the gelation required intermolecular interactions such as hydrogen bonding and π–π interaction. The absolute configuration of the gelled tri(PAAm) was determined using DFT calculation by comparing the calculated CD spectrum with the experimentally measured spectrum. This study presents the first report on constructing a stereoisomer library for vinyl oligomers. Stereoisomer libraries, which can be easily prepared *via* one-pot radical polymerization, are promising new modalities for supramolecular chemistry, highly functional drugs, drug delivery media and photoelectromagnetic materials such as chiral emissive dyes and chiral magnetic materials.

## Author contributions

Y. H. conceived the project. Y. N., H. I., S. F., S. A., S. I., H. T., T. Y., K. W., and S. T. performed the experimental studies. T. T. performed the computational studies. T. O, Y. M., and Y. H. supervised the research. Y. N. and H. I. wrote the original draft of the manuscript which was edited by all authors.

## Conflicts of interest

There are no conflicts to declare.

## Supplementary Material

SC-016-D5SC00612K-s001

## Data Availability

The authors confirm that the data supporting the findings of this study are available within the article and its ESI.†
