# Supplementary material for: Stereoisomer library prepared via controlled radical polymerization: isolation, structural identification and discovery of stereospecific gelation behaviour of tri(N-phenyl acrylamide)
Source: Chem Sci. 2025 May 20;16(25):11475–80. doi: 10.1039/d5sc00612k (PMC12109607; doi:10.1039/d5sc00612k)
Supplement: SC-016-D5SC00612K-s001 [file SC-016-D5SC00612K-s001.pdf]

Supplementary Information (SI) for Chemical Science.
This journal is © The Royal Society of Chemistry 2025

# Supporting Information

## Stereoisomer library prepared via controlled radical polymerization: Isolation, structural identification, and discovery of stereospecific gelation behavior of tri(*N*-phenyl acrylamide)

Yukiko Nagai[a, ‡], Hinako Iwamoto[b, ‡], Satoki Fukuda[b], Sotaro Akashi[b], Shota Iseri[b], Hayato Tada[a], Tomohiro Yamanaka[a], Konosuke Wada[a], Sotaro Tsuji[a], Toshikazu Ono[a][d], Yoshiko Miura[b], Tohru Taniguchi[c] and Yu Hoshino[a][d]*

‡These authors contributed equally.

[a] Department of Applied Chemistry, Graduate School of Engineering, Kyushu University, 744 Motooka Nishi-ku, Fukuoka 819-0395, JAPAN

[b] Department of Chemical Engineering, Graduate School of Engineering, Kyushu University, 744 Motooka Nishi-ku, Fukuoka 819-0395, JAPAN

[c] Department of Advanced Transdisciplinary Sciences, Faculty of Advanced Life Science, Hokkaido University, Kita 10, Nishi 8, Kita-ku, Sapporo 060-0810, JAPAN

[d] Center for Molecular systems (CMS), Kyushu University, 744 Motooka Nishi-ku, Fukuoka 819-0395, JAPAN

Section S1 Materials
Section S2 Characterization
Section S3 DFT calculation of the CD spectrum
Section S4 Synthesis of (R)-2-cyanopropan-2-yl (2,3-dihydroxypropyl) carbonotrithioate (CPDOTT(R))
Section S5 Preparation of discrete PAAm oligomers
Section S6 Analysis of diastereomers of tri(PAAm)
Section S7 Chiral separation of tri(PAAm)
Section S8 Gelation of tri(PAAm)
Reference

**Section S1 Materials**

*N*-Phenylacrylamide and sodium hydride (NaH) was purchased from Tokyo Chemical Industry (Tokyo, Japan). 2,2'-azobis(isobutyronitrile) (AIBN, 98%), was purchased from Wako Pure Chemical Industries (Osaka, Japan). Sodium sulfate ($Na_2SO_4$, 99%) and sodium chloride (NaCl, 99%) were purchased from Kanto Chemical (Tokyo, Japan). R-2-Cyanopropan-2-yl 2,3-propanediyl trithiocarbonate (CPDOTT (R)) was synthesized as in Section S3. Other commercial solvents were purchased from Kanto Chemical (Tokyo, Japan). Sodium hydroxide solution (0.01 N) was purchased from Sigma-Aldrich Japan (Tokyo, Japan). 2,2-Azobis(isobutyronitrile) was purified by recrystallization from MeOH and dried in vacuo at room temperature. Cation exchange resin (Muromac C1002−H) and was purchased from Muromachi Technos Co., Ltd. Water used in this experiment was prepared using a Direct-Q Ultrapure Water System (Merck, Limited).

**Section S2 Characterization**

Proton nuclear magnetic resonance ($^1H$ NMR) spectra were recorded in $CD_3OD$ on a JEOL-ECP400 spectrometer. Automated flash column chromatography was performed on a Biotage Isolera One ISO-1SW instrument, equipped with a Biotage SNAP Ultra C18 column cartridge (50 g), and eluted with Hexane and EtOAc. For ultra-performance liquid chromatography-mass spectrometry (UPLC-MS), an ACQUITY Sample Manager FTN-H (Waters, Waters Corporation, Massachusetts, USA), ACQUITY Quaternary Solvent Manager (Waters), ACQUITY UPLC® BEH C18 1.7 µm 21 × 50 mm column (Waters), CHIRALPAK IG 5 µm 4.6 ×150 mm (DAICEL), ACQUITY Column Manager (Waters), PDA eλ Detector (Waters), and ACQUITY QDA detector (Waters) were used. The data were analyzed by using Empower SOFTWARE (Waters). To analyze the oligomer, the samples were dissolved in MeOH and analyzed by UPLC-MS at room temperature. The column was equilibrated with MilliQ water (containing 0.1% formic acid) at a flow rate of 0.3 mL/min. Subsequently, 10 µL of sample was injected by ACQUITY Sample Manager, and elution was carried out by increasing the percentage of MeOH in the eluent from 0 to 100%. Detection was performed using a PDA eλ detector (wavelength = 310, 250 nm) and an ACQUITY QDA detector (ionization mode: ESI positive). A cation exchange resin (Muromac C1002−H) was pretreated with 1 M HCl then washed with Milli-Q water. Recycled preparative HPLC was performed on LaboACE LC-7080(JAI), equipped with a JAIGEL-ODS-AP-50 50 ×250 mm (JASCO) and eluted with water and MeOH. For high-performance liquid chromatography (HPLC), LC-2000Plus (JASCO) (JASCO PU-986 Intelligent Prep. Pump, JASCO MX-2080-32 Dynamic Mixer, JASCO DG-980-50 degasser, JASCO LC-Netll/ADC, JASCO MD-4010 Photo Diode Array Detector CHIRALPAK AD-H (Φ10×250 mm and Φ30×250 mm) (DAICEL), CHIRALCEL OX-H (Φ4.6×250 mm and Φ30×250 mm) (DAICEL) were used. Circular dichroism spectrometer (CD) was performed on j-1500 (JASCO) in MeOH. Atomic force microscopy (AFM) was conducted on Agilent 5500.

**Section S3 DFT calculation of the CD spectrum**

Preliminary MMFF conformational search of the arbitrarily chosen $R_a$,$S_c$,$S_e$ stereoisomer was carried out by SPARTAN'20 software. [1] The obtained 57 geometries within 8.0 kcal/mol from the most stable were optimized at DFT/B3LYP/6-31G(d) on SPARTAN'20. The resultant 17 conformers within 3.0 kcal/mol from the most stable were further optimized at DFT/M06-2X/6-311G(2df,p) with PCM for methanol on Gaussian16, [2] which led to 4 stable conformers in a 1.75 kcal/mol energy window. The ECD spectra of the resultant conformers were calculated at DFT/M06-2X/6-311G(2df,p) with PCM for methanol. The first 100 singlet → singlet electronic transitions of each conformer were calculated and then converted to ECD spectra on GaussView 6 software using Gaussian band shapes with 0.310 eV half-width at half height. Each spectrum was averaged based on the Boltzmann population at 298 K. The calculated wavelengths were manually shifted to longer wavelength by 20 nm.

**Section S4 Synthesis of (R)-2-cyanopropan-2-yl (2,3-dihydroxypropyl) carbonotrithioate (CPDOTT(R))**

Thioacetic acid (4.91 g, 64.5 mmol, 1.24 eq) was added to (R)-(+)-glycidol (optical purity ee 98%, 3.9 g, 52 mmol, 1 eq) in an ice bath and stirred at room temperature for 24 hours. After completion of the reaction, residual thioacetic acid was removed by an evaporator, and the product was purified by low-pressure flash column chromatography (Hexane/EtOAc) and dried in vacuum overnight (Yield: 9.27 g, 95.8 %) (**Compound 1**). $^1$H NMR ($CDCl_3$/TMS) in ppm:2.36 (s, 3 H), 3.00 (dd, *J*=6.8, 14.4 Hz, 1 H),3.10 (dd, *J*=6.0, 14.4 Hz, 1 H), 3.56 (dd, *J*=5.2, 11.2 Hz, 1 H), 3.66 (dd, *J*=3.6, 11.6 Hz, 1 H), 3.80 (m, 1 H) (Figure S1).

**Compound 1** (Mw 150.1) (9.27 g, 61.8 mmol, 1 eq) and (+)-10-Camphorsulfonic Acid (4.11 g, 0.018 mmol) dissolved in 2,2-dimethoxypropane (150 mL, 1.42 mmol, 23 eq) and stirred at room temperature for 3 hours. After completion of the reaction, dichloromethane (300 mL) was added, and the mixture was separated with an aqueous sodium hydrogencarbonate solution (30 g/L) and milliQ water three times each. Then, it was separated once with saturated NaCl aqueous solution, dehydrated by adding $Na_2SO_4$, vacuum filtered, concentrated under reduced pressure with an evaporator, and purified by low-pressure flash column chromatography (Hexane/EtOAc) (Sfar Silica HCD Duo 20 μm 50 g). Then vacuum dried overnight (Yield: 7.74 g, 65.9 %) (**Compound 2**). $^1$H NMR ($CDCl_3$/TMS) in ppm: 1.33 (s, 3 H), 1.42 (s, 3 H), 2.35 (s, 3 H), 3.06 (m, 1 H), 3.62 (dd, *J*=8.4, 6.0 Hz, 1 H), 4.06 (dd, *J*=8.4, 6.0 Hz, 1 H), 4.22 (m, 1 H) (Figure S2).

**Compound 2** (Mw 190) (3.87 g, 20.3 mmol, 1 eq) was dissolved in MeOH (111 mL), sodium methoxide (1.20 g, 0.2 M) was added, and the mixture was stirred at room temperature for 2 hours. After completion of the reaction, cation exchange resin (6.27 g) was added and stirred. After confirming that it was neutral with pH test paper, it was vacuum-filtered and concentrated under reduced pressure using an evaporator (Yield: 2.64 g, 88 %) (**Compound 3**). $^{1}H$ NMR ($CDCl_3$/TMS) in ppm:1.35 (s, 3 H), 1.42 (s, 3 H), 2.60 (m, 1 H), 2.73 (m, 1 H), 3.75 (dd, $J$=8.4, 6.0 Hz, 1 H), 4.10 (dd, $J$=8.4, 6.4 Hz, 1 H), 4.19 (m, 1 H) (Figure S3).

After the two-necked eggplant flask was heated with a heat gun and purged with nitrogen, NaH (0.70 g, 29.2 mmol, 1.67 eq) was put into the flask and purged with nitrogen again. After that, dry $Et_2O$ (95.1 mL) and **Compound 3** (2.64 g, 17.8 mmol, 1 eq) were sequentially added dropwise into the flask using a dropping funnel, and the mixture was stirred in ice for 15 minutes. $CS_2$ (2.22 g, 29.2 mmol, 1.67 eq) was then added dropwise using a dropping funnel in an ice bath and stirred for 2 hours. After a period of time, the solution was concentrated under reduced pressure using an evaporator to remove residual $CS_2$. After replacing the inside of the flask with nitrogen again, dry $Et_2O$ (95.6 mL) and $I_2$ (4.5 g, 17.8 mmol, 1 eq) were added, and the mixture was stirred at room temperature for 1 hour. Then, it was washed three times with 100 mL of sodium thiosulfate aqueous solution (30 g/L) and three times with 100 mL of water. The resulting organic layer was dehydrated with sodium sulfate, filtered under vacuum, and concentrated under reduced pressure using an evaporator. It was then vacuum dried overnight (Yield: 2.81 g, 70 %) (**Compound 4**). $^{1}H$ NMR ($CDCl_3$/TMS) in ppm:1.32 (s, 3 H), 1.41 (s, 3 H), 3.54 (d, $J$=5.6 Hz, 2 H), 3.66 (dd, $J$=8.8, 6.0 Hz, 1 H), 4.07 (dd, $J$=8.8, 6.0 Hz 1 H), 4.36 (m, 1 H) (Figure S4).

In a two-necked flask, prepared **compound 4** (2.81 g, 6.30 mmol, 1 eq) and AIBN (2.07 g, 12.6 mmol, 2 eq) were dissolved in EtOAc (47 mL), and nitrogen was bubbled for 30 minutes. and refluxed under nitrogen for 18 hours. Then, it was dissolved in a mixed solution of 1 M HCl (6.7 mL) and dioxane (60.2 mL), and stirred in an oil bath at 60°C for 1 hour. After that, $NaHCO_3$ aqueous solution was added to neutralize (pH = around 6.0). At this time, the decomposition of the trithio group occurs when it becomes basic (pH > 7), and the decomposition of the compound occurs when it is concentrated in an acidic state, so the operation was performed carefully. After the neutral solution was concentrated under reduced pressure using an evaporator, it was purified by low-pressure flash column chromatography (Hexane/EtOAc) and vacuum-dried overnight (Yield:1.10 g, 35 %) (**Compound 5**: CPDOTT (R)). $^{1}H$ NMR ($CD_3OD$/TMS) in ppm:1.88 (s, 6 H), 3.42 (dd, $J$=16, 8 Hz, 1 H), 3.57 (d, $J$=3.6 Hz, 2 H), 3.71 (dd, $J$=12, 8 Hz, 1 H), 3.88 (m, 1 H) (Figure S5). Furthermore, using UPLC, CHIRAL PAK IG was run under reversed-phase conditions (MeOH 100%), and compared with CPDOTT (racemate), stereoselective It was confirmed that it was a synthetic product (Figure S6).

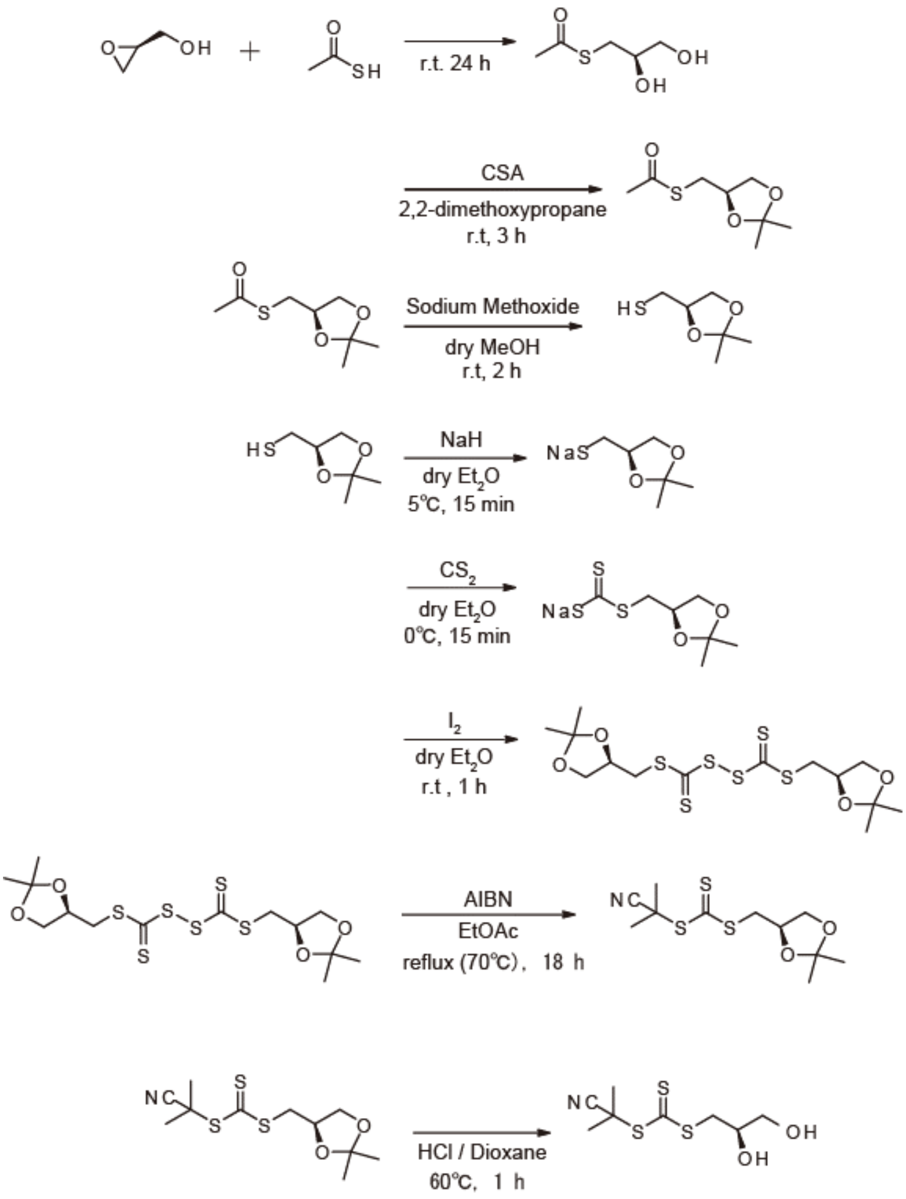


Scheme S1. Synthetic scheme of CPDOTT (R).

| | a1 | a2 | b | c | c1 | d |
|---|---|---|---|---|---|---|
| Integral | 0.92 | 0.92 | 1.00 | 0.94 | 0.93 | 3.02 |

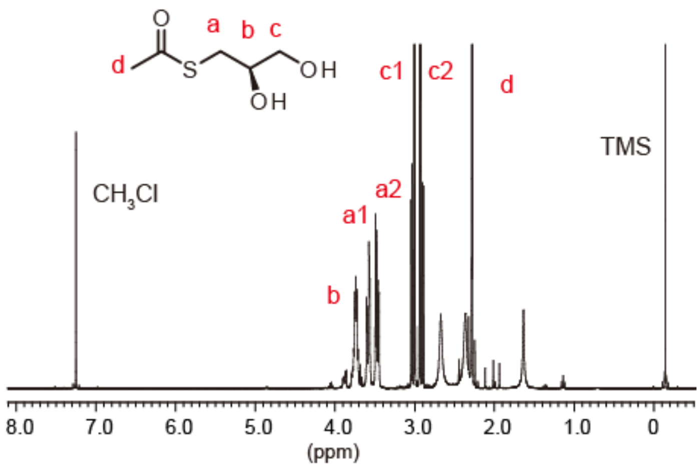


Figure S1. $^{1}$H NMR spectra of **compound 1** in $CDCl_3$.

| | a1 | a2 | b | c | d | e1 | e2 |
|---|---|---|---|---|---|---|---|
| Integral | 1.02 | 0.98 | 1.00 | 2.08 | 3.18 | 3.35 | 3.05 |

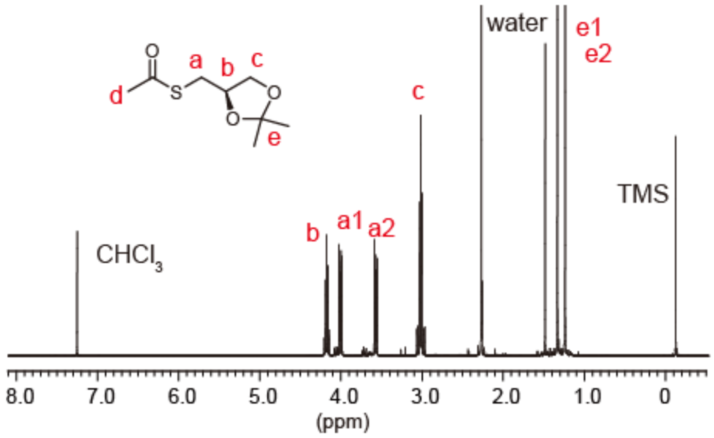


Figure S2. $^{1}$H NMR spectra of **compound 2** in $CDCl_3$.

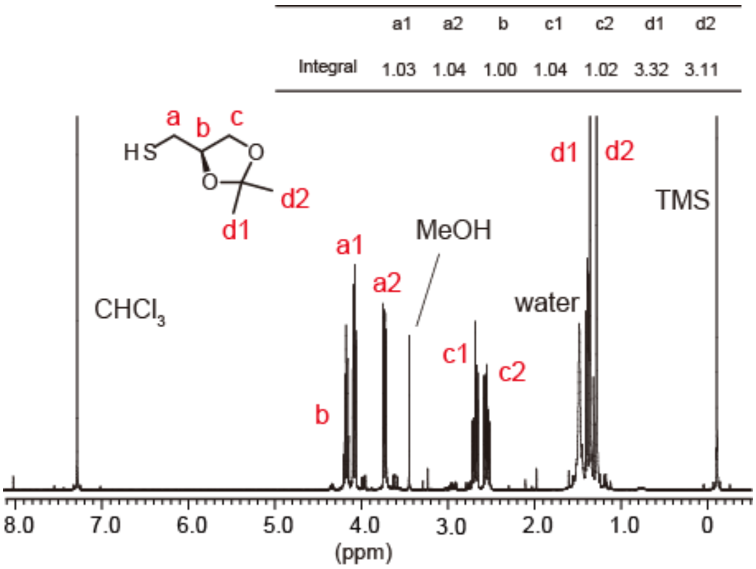


Figure S3. $^{1}$H NMR spectra of **compound 3** in $CDCl_3$.

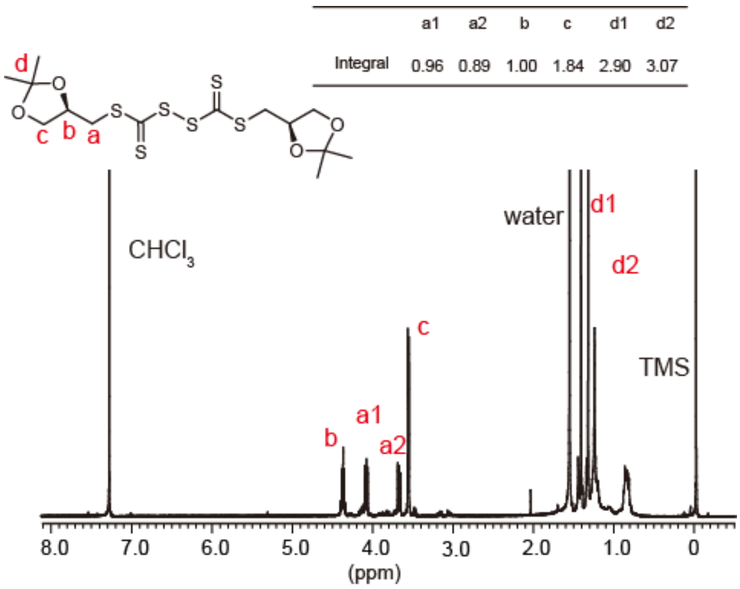


Figure S4. $^{1}$H NMR spectra of **compound 4** in $CDCl_3$.

| | a1 | a2 | b | c | d |
|---|---|---|---|---|---|
| Integral | 1.03 | 1.05 | 1.07 | 2.12 | 6.00 |

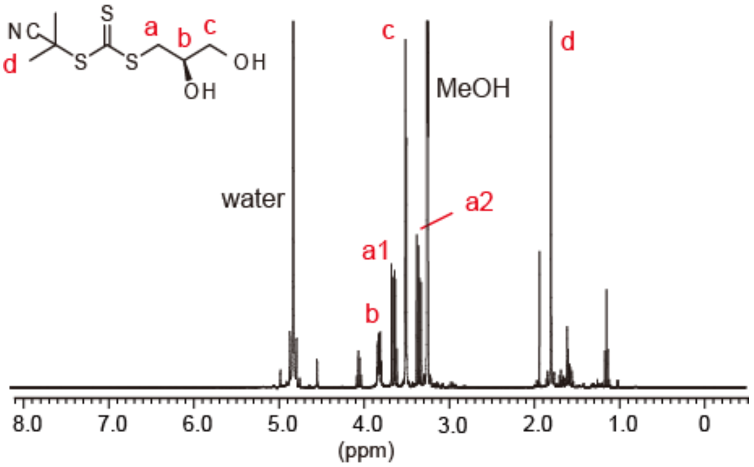


Figure S5. $^{1}$H NMR spectra of **compound 5** (CPDOTT(R)) in $CD_3OD$.

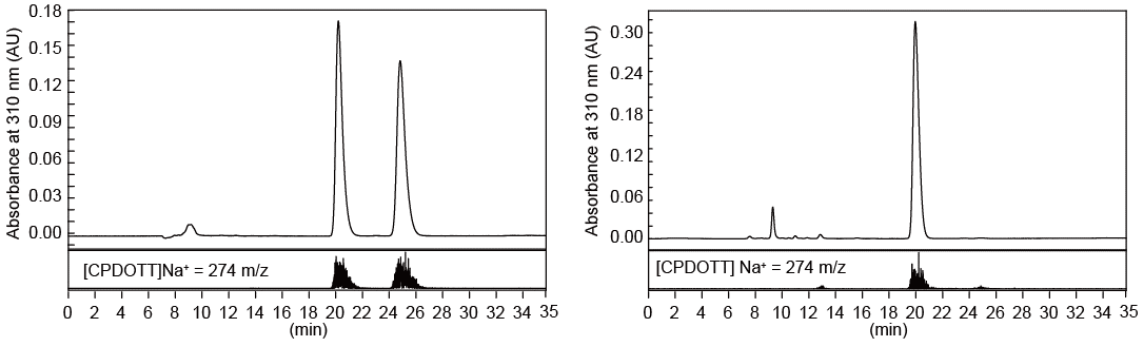


Figure S6. Chromatogram (upper) and mass spectrum (lower) of CPDOTT (racemate) (left), CPDOTT (R) (right).

**Section S5 Preparation of discrete PAAm oligomers**

The monomer (*N*-Phenylacrylamide, 2.03 g, 1.85 M) was dissolved in 1,4-dioxane (7.45 mL) with chain transfer agent (CPDOTT (R), 1.38 g, 0.74 M) and the radical initiator (AIBN, 0.180 g, 0.15 M). The mixture was degassed by repeated freeze-thaw cycles (three times). After degassing, the reaction was carried out at 70°C for 6 h in an oil-bath. The reaction was stopped by exposure to air (Scheme S2). The solutions before and after polymerization were analyzed by $^{1}$H NMR, and the conversion was calculated from the percentage decrease of the vinyl groups (conversion ≥ 99%) (Figure S7). The polymerization-terminated liquid was concentrated by an evaporator and vacuum-dried overnight to

remove 1,4-dioxane. The product was isolated by reverse-phase high-performance liquid chromatography (HPLC). A C18 column was used for separation, and the liquid obtained after equilibration and vacuum drying was loaded onto the column. MilliQ water/MeOH was used as the mobile phase, and the sample was collected at an absorption wavelength of 250 nm (Figure S8).

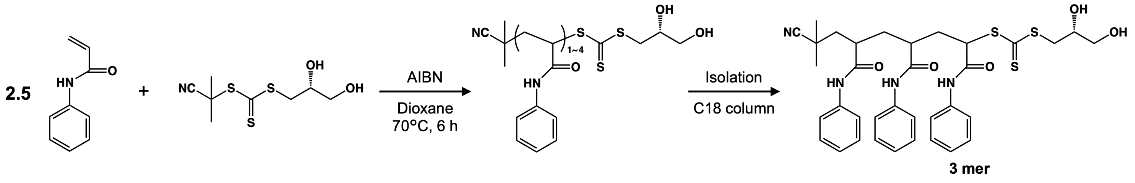


Scheme S2. Preparation of discrete PAAm oligomers.

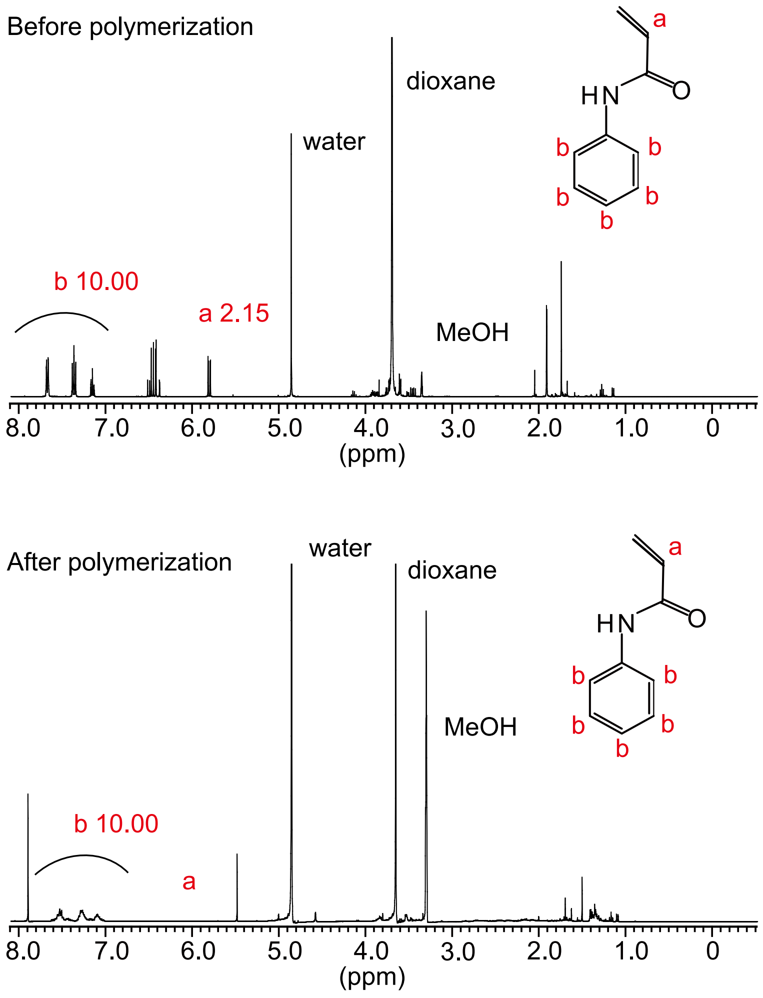


Figure S7. $^{1}$H NMR spectra of PAAm before and after polymerization in $CD_3OD$.

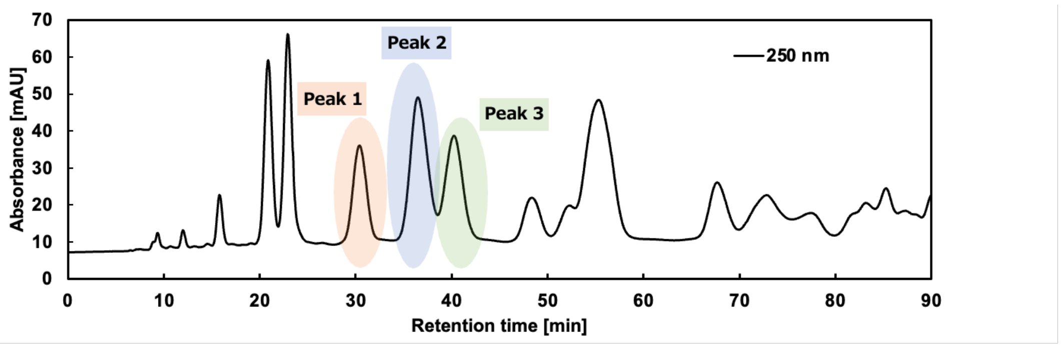


Figure S8. Chromatogram for separation of discrete PAAm oligomers. Tri(PAAm) has the three diastereomer peaks.

**Section S6 Analysis of diastereomers of tri(PAAm)**

The three diastereomers of the tri(PAAm) separated in section S4 were separated in ascending order of elution time, called Peak 1, Peak 2, and Peak 3, respectively (Figure 8), and their molecular weights were measured (Figure S9, left). As a result, the molecular weight of tri(PAAm) ($[M + Na]^+$) was confirmed in all peaks. In addition, there was a shift in the proton peak of the main chain as indicated by $^1$H NMR analysis of each of them (Figure S9, right).

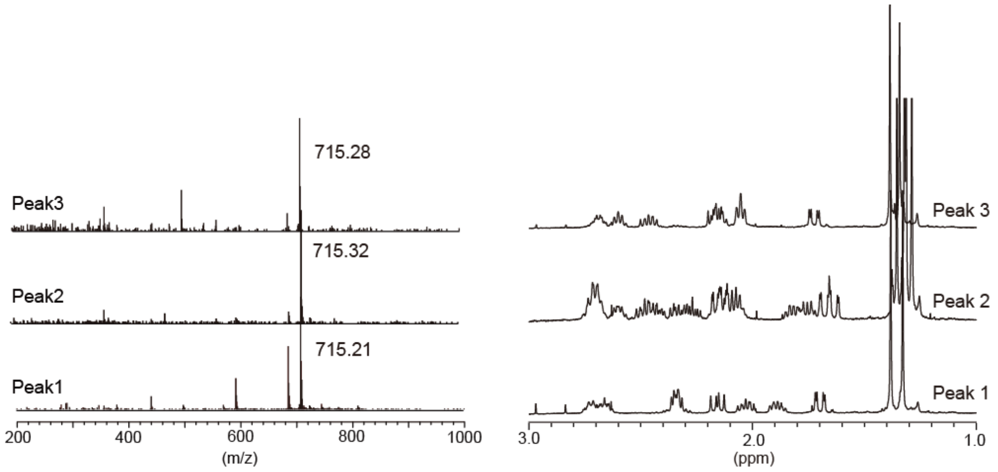


Figure S9. MS spectra (left) and $^1$H NMR spectra (right) of the diastereomers of tri(PAAm).

**Section S7 Chiral separation of tri(PAAm)**

Prepare a sample solution for separation by dissolving each diastereomer separated in section S5 in Hexane/EtOH = 70/30 to a sample concentration of 5 mg/mL and passing through a solvent-based syringe filter (0.45 μm). A chiral column (Daicel, CHIRAL PAK AD-H, Φ10 mm×250 mm, after TFA treatment) connected to HPLC (JASCO) was used for separation of tri(PAAm) Peak 1 and Peak 2 (Sample loop: 1 mL) (Figure S10, 11). Equilibration was carried out by passing a mobile phase (Hexane/EtOH=70/30) at 9 mL/min for 30 minutes. After equilibration, 400 μL of the sample solution was injected, and separation was performed with a mobile phase (Hexane/EtOH=70/30), a flow rate of 9 mL/min, and a column temperature of 40°C. For separation of tri(PAAm) Peak 3, chiral column (Daicel, CHIRALCEL OX-H, Φ30 mm×250 mm) connected to HPLC (JASCO) was used (Sample loop: 1 mL) (Figure S12). Equilibration was carried out by passing a mobile phase (MeCN) at 9 mL/min for 30 minutes. After equilibration, 800 μL of the sample solution was injected, and separation was performed with a mobile phase (MeCN), a flow rate of 9 mL/min, and a column temperature of 25°C. Each fraction after separation was subjected to $^{1}$H NMR and MS spectrum measurement. MS confirmed the molecular weight of tri(PAAm) in all fractions (Figure S13, 14, 15, 16, 17, 18, 19, 20). In $^{1}$H NMR, each proton was assigned to the proton peak of the main chain based on JH and splitting (Figure S13, 14, 15, 16, 17, 18, 19, 20). The relative configuration was assigned from the deviation of the main chain proton peak (Figure S21). The stereoregular tri(PAAm) were characterized by CD spectroscopy using MeOH as a solvent (adjusted the absorbance at 0.5). The mirror images in CD spectra of each enantiomeric pair were observed in solutions (Figure S23). In addition, the CD spectra of two enantiomeric pairs when adjusting the absorbance at 0.2, 0.5, and 1 (Figure S24), and no concentration dependency was observed.

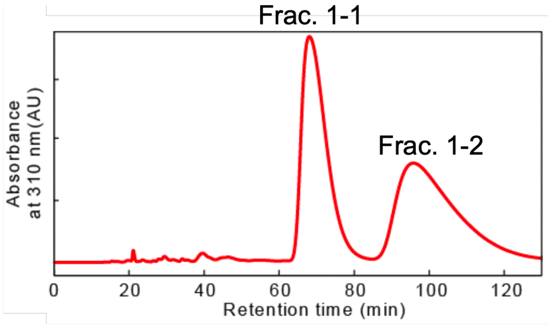


Figure S10. Chromatogram of Peak 1 (Frac. 1-1 (69 min), Frac. 1-2 (98 min)).

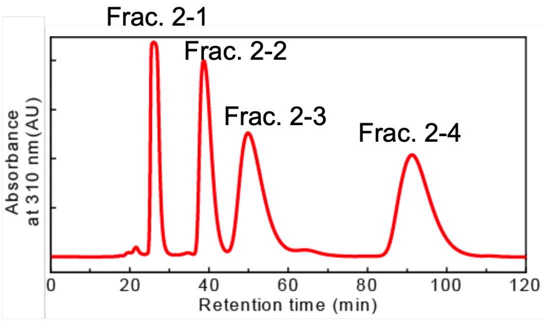


Figure S11. Chromatogram of Peak 2 (Frac. 2-1 (27 min), Frac. 2-2 (39 min), Frac. 2-3 (50 min), Frac. 2-4 (92 min)).

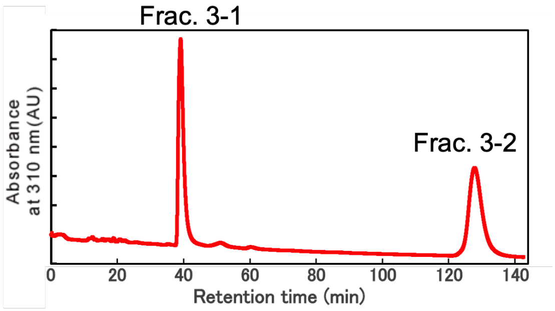


Figure S12. Chromatogram of Peak 3 (Frac. 3-1 (39 min), Frac. 3-2 (128 min)).

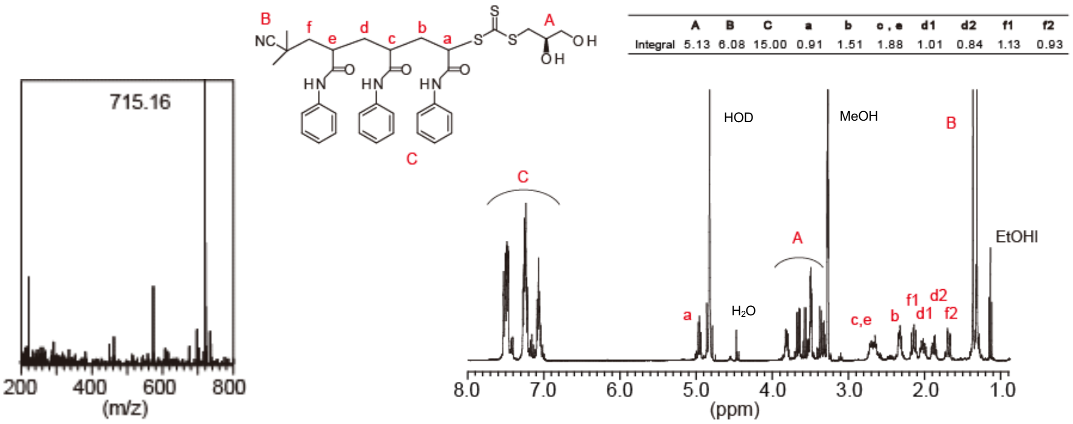


Figure S13. MS spectrum (left) and $^1$H NMR spectrum (right) of Frac. 1-1.

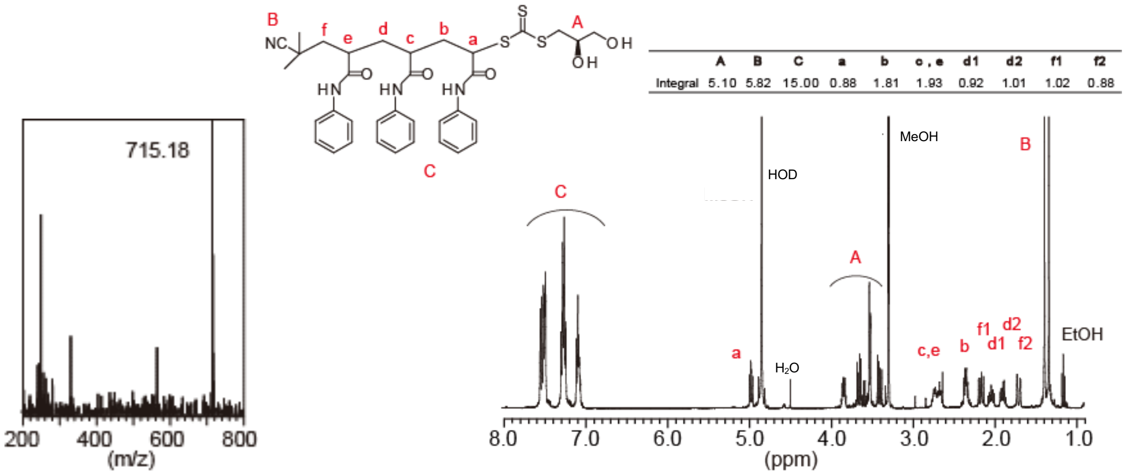


| | A | B | C | a | b | c , e | d1 | d2 | f1 | f2 |
|---|---|---|---|---|---|---|---|---|---|---|
| Integral | 5.10 | 5.82 | 15.00 | 0.88 | 1.81 | 1.93 | 0.92 | 1.01 | 1.02 | 0.88 |

Figure S14. MS spectrum (left) and $^{1}$H NMR spectrum (right) of Frac. 1-2.

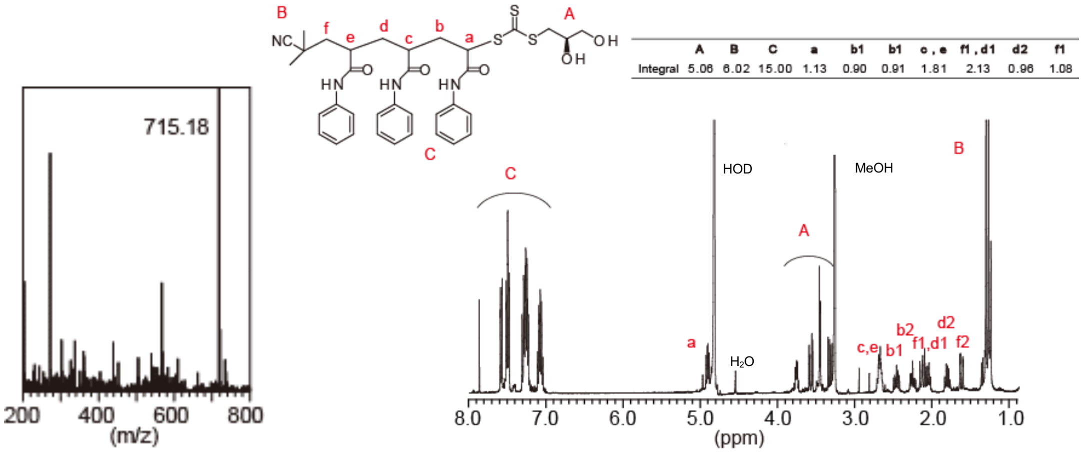


| | A | B | C | a | b1 | b1 | c , e | f1 , d1 | d2 | f1 |
|---|---|---|---|---|---|---|---|---|---|---|
| Integral | 5.06 | 6.02 | 15.00 | 1.13 | 0.90 | 0.91 | 1.81 | 2.13 | 0.96 | 1.08 |

Figure S15. MS spectrum (left) and $^{1}$H NMR spectrum (right) of Frac. 2-1.

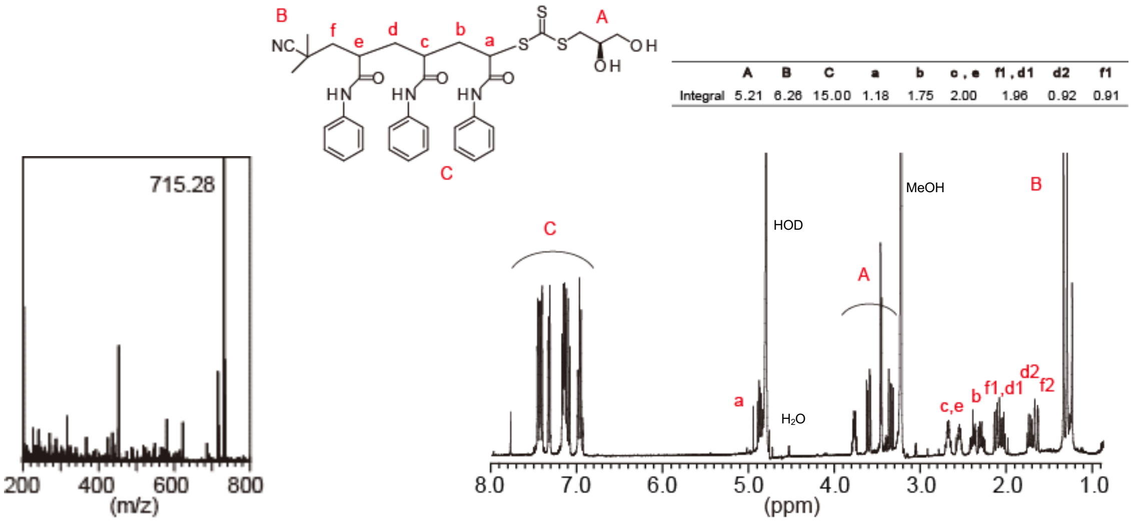


| | A | B | C | a | b | c , e | f1 , d1 | d2 | f1 |
|---|---|---|---|---|---|---|---|---|---|
| Integral | 5.21 | 6.26 | 15.00 | 1.18 | 1.75 | 2.00 | 1.96 | 0.92 | 0.91 |

Figure S16. MS spectrum (left) and $^1$H NMR spectrum (right) of Frac. 2-2.

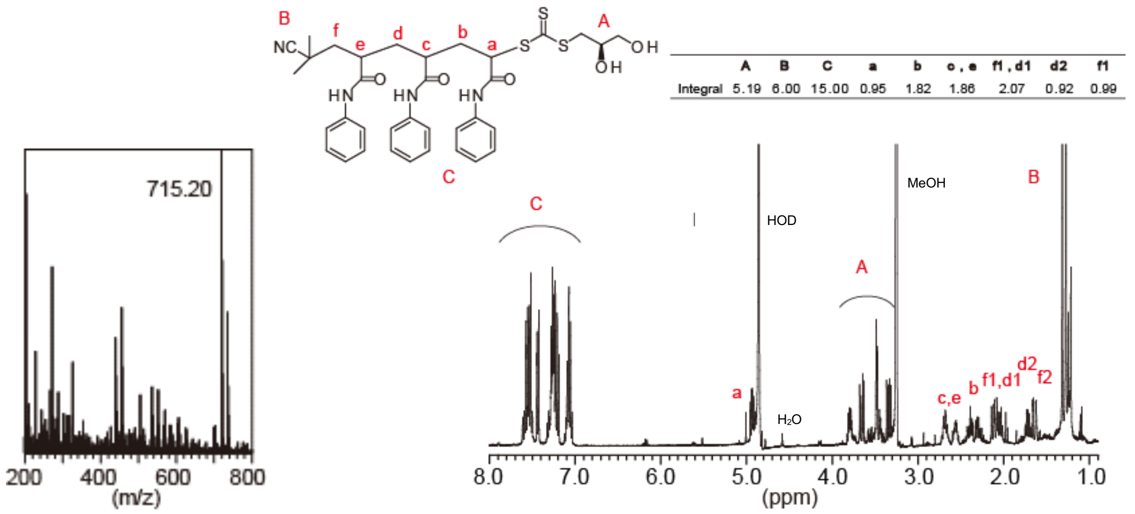


| | A | B | C | a | b | c , e | f1 , d1 | d2 | f1 |
|---|---|---|---|---|---|---|---|---|---|
| Integral | 5.19 | 6.00 | 15.00 | 0.95 | 1.82 | 1.86 | 2.07 | 0.92 | 0.99 |

Figure S17. MS spectrum (left) and $^1$H NMR spectrum (right) of Frac. 2-3.

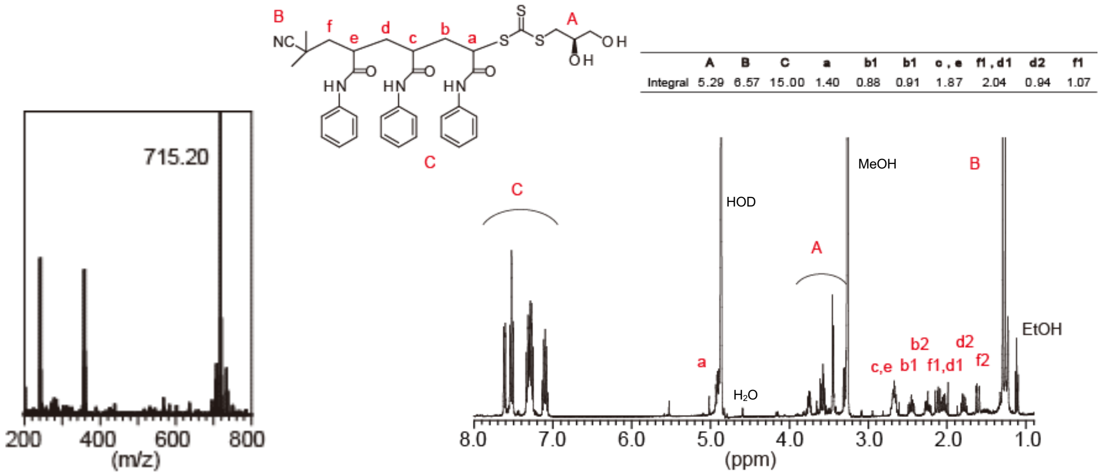


Figure S18. MS spectrum (left) and $^1$H NMR spectrum (right) of Frac. 2-4.

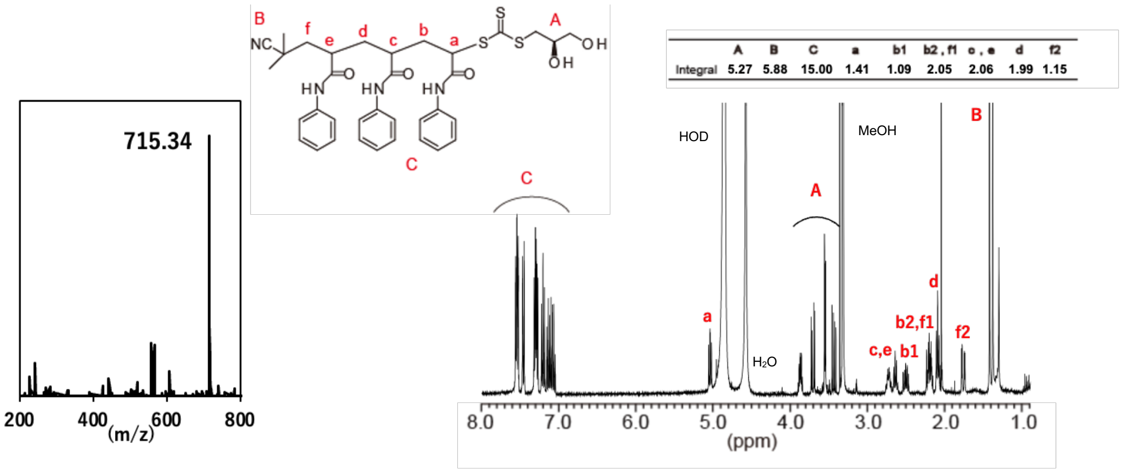


Figure S19. MS spectrum (left) and $^1$H NMR spectrum (right) of Frac. 3-1.

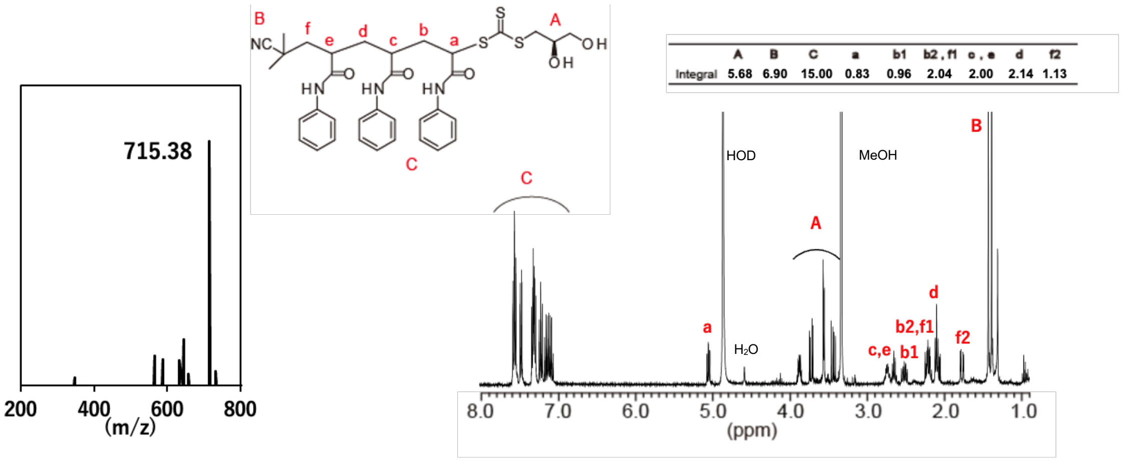


Figure S20. MS spectrum (left) and $^{1}$H NMR spectrum (right) of Frac. 3-2.

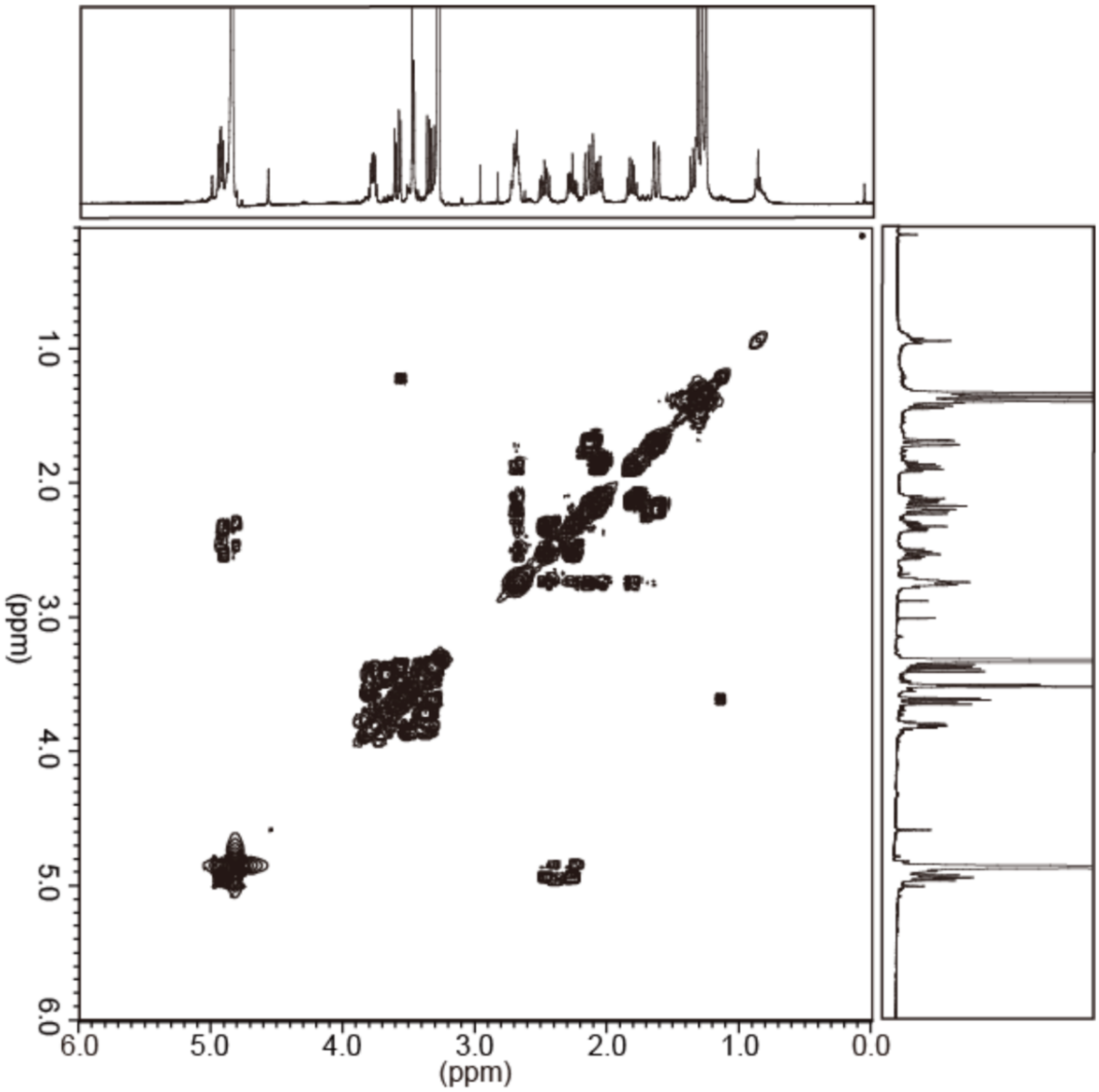


Figure S21. $^{1}$H-$^{1}$H COSY spectrum of tri(PAAm) in Frac. 2-1.

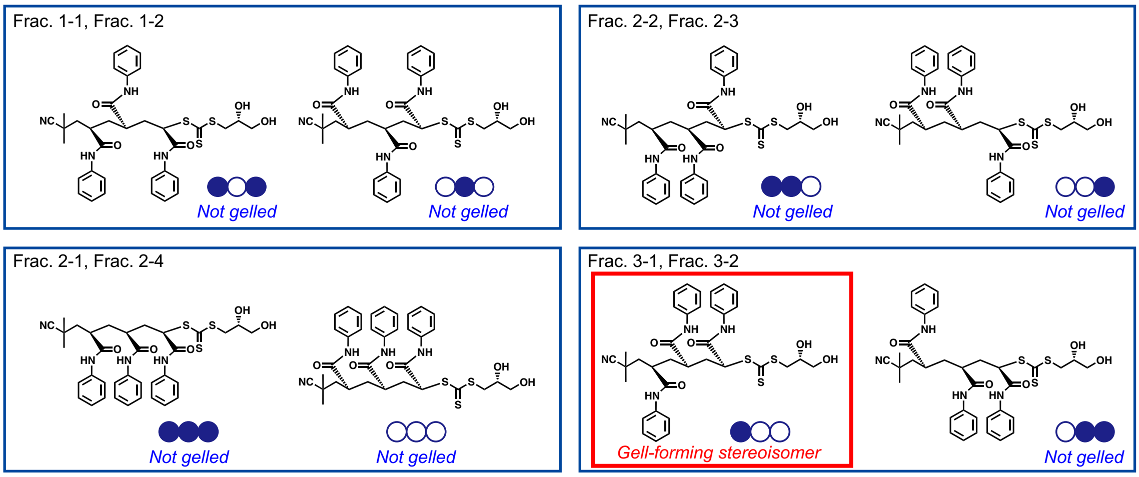


Figure S22. The relative configurations of each fraction and their gelation behavior in chloroform.

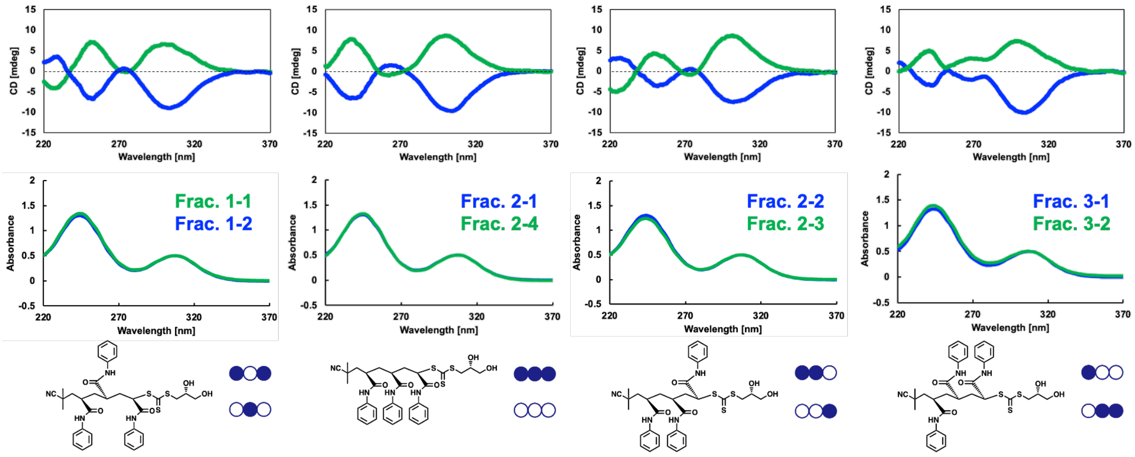


Figure S23. CD and absorption spectra of stereoisomers with an enantiomeric relationship in the asymmetric carbons on the tri(PAAm) structure, (a) Frac. 1-1 and 1-2, (b) Frac. 2-1 and 2-4, (c) Frac. 2-2 and 2-3, (d) Frac. 3-1 and 3-2.

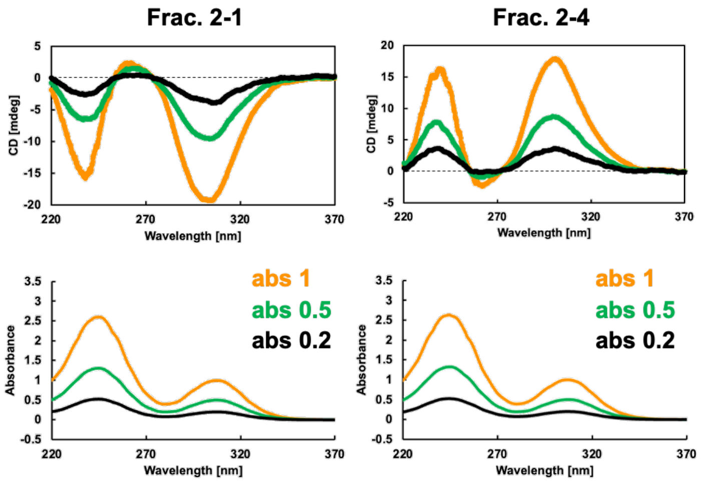


Figure S24. Concentration-dependent CD and absorption spectra of the enantiomeric pair, Frac. 2-1 and 2-4, when adjusting the absorbance 0.2 (black line), 0.5 (green line), and 1 (orange line).

**Section S8 Gelation of tri(PAAm)**

Gelation of each diastereomer pair and stereoregular tri(PAAm) were investigated using chloroform as a solvent, adjusting the concentration at 15, 20, 30, and 40 mg/mL. After standing at 4°C for 24 h, the gelation of the stereoregular Frac. 3-1 was observed at 40 mg/mL. AFM image was obtained with dry condition in air at room temperature.

Table S1. Gelation of stereoregular tri(PAAm).

| | 15 mg/mL | 20 mg/mL | 30 mg/mL | 40 mg/mL |
|---|---|---|---|---|
| **P1 Frac. 1-1** | 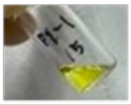 liquid | 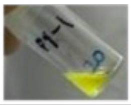 liquid | 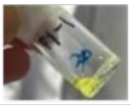 liquid | 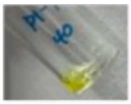 liquid |
| **P1 Frac. 1-2** | 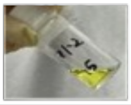 liquid | 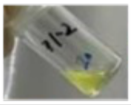 liquid | 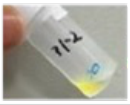 liquid | 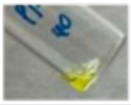 liquid |
| **P2 Frac. 2-1** | 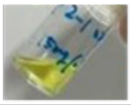 liquid | 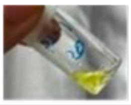 liquid | 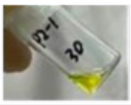 liquid | 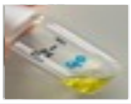 liquid |
| **P2 Frac. 2-2** | 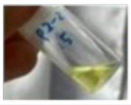 liquid | 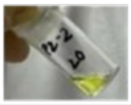 liquid | 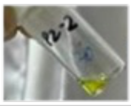 liquid | 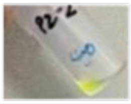 liquid |
| **P2 Frac. 2-3** | 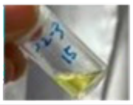 liquid | 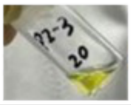 liquid | 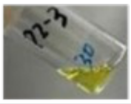 liquid | 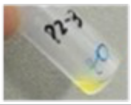 liquid |
| **P2 Frac. 2-4** | 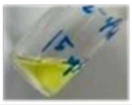 liquid | 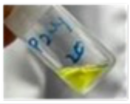 liquid | 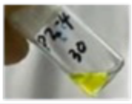 liquid | 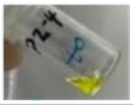 liquid |
| **P3 Frac. 3-1** | 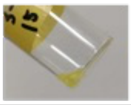 liquid | 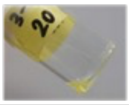 liquid | 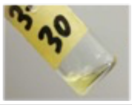 liquid | 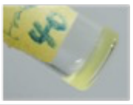 gel |
| **P3 Frac. 3-2** | 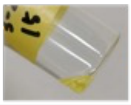 liquid | 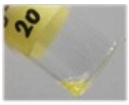 liquid | 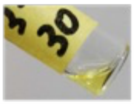 liquid | 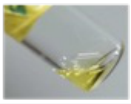 liquid |

Table S2. Gelation of the mixture of two stereoisomers with an enantiomeric relationship in the asymmetric carbons on the tri(PAAm) structure.

| | 15 mg/mL | 20 mg/mL | 30 mg/mL | 40 mg/mL |
|---|---|---|---|---|
| **Frac. 1-1**<br>**Frac. 1-2** | 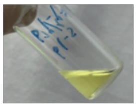 liquid | 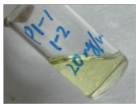 liquid | 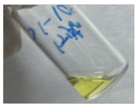 liquid | 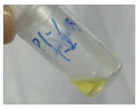 liquid |
| **Frac. 2-1**<br>**Frac. 2-4** | 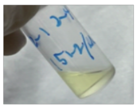 liquid | 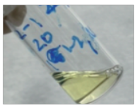 liquid | 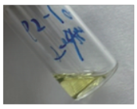 liquid | 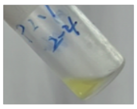 liquid |
| **Frac. 2-2**<br>**Frac. 2-3** | 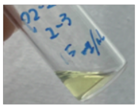 liquid | 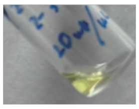 liquid | 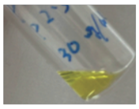 liquid | 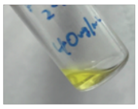 liquid |
| **Frac. 3-1**<br>**Frac. 3-2** | 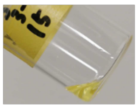 liquid | 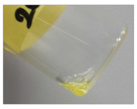 liquid | 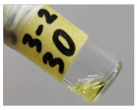 liquid | 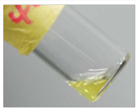 liquid |

Table S3. Gelation of diastereomer Frac. 3-1 in different solvents.

| Solvent | Dielectric constant | Frac. 3-1 40 mg/mL |
|---|---|---|
| Hexane | 1.9 | 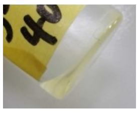 N/A |
| Diethyl ether | 4.3 | 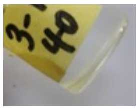 N/A |
| Chloroform | 4.8 | 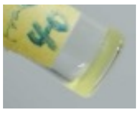 gel |
| EtAOc | 6.0 | 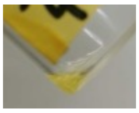 liquid |
| THF | 7.6 | 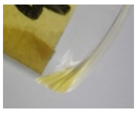 liquid |
| NMP | 32.0 | 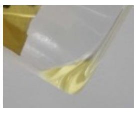 liquid |
| EtOH | 24.6 | 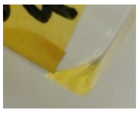 liquid |
| MeOH | 32.0 | 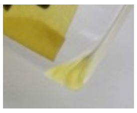 liquid |
| DMF | 36.7 | 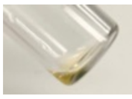 liquid |
| Chloroform + benzene (10%) | – | 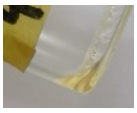 liquid |

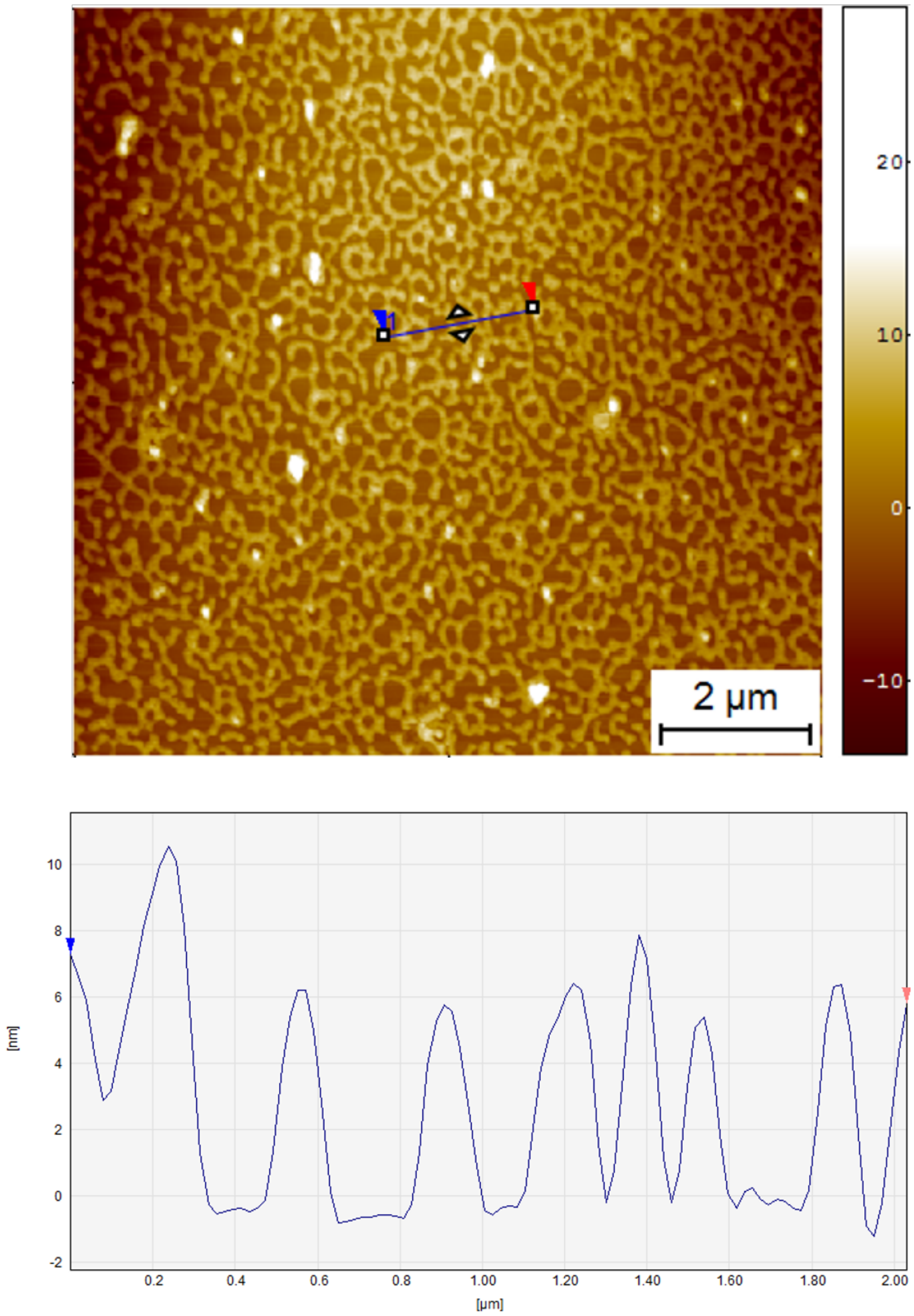


Figure S25. AFM image (up) and the histogram (bottom) of Frac. 3-1.

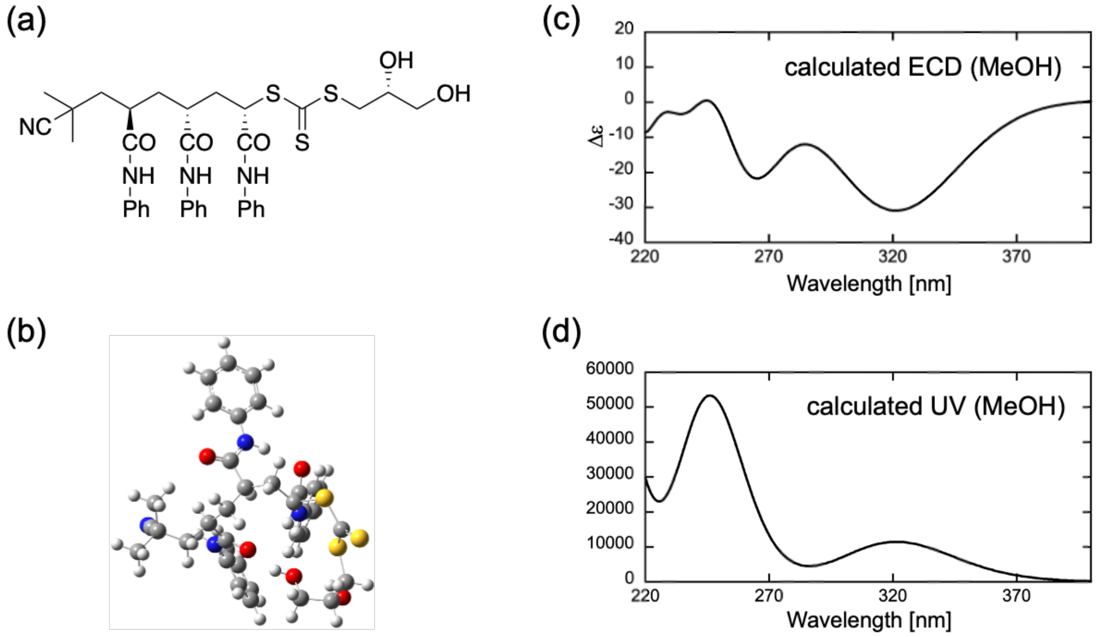


Figure S26. The chemical structure of (a) the tri(PAAm) for DFT calculation and (b) its stable conformer. The calculated (c) CD spectrum and (d) absorption spectrum of the tri(PAAm) shown in Figure S26a.

**Feference**

[1] Spartan'20, Wavefunction, Inc., Irvine, CA.

[2] R. C. Gaussian 16, M. J. Frisch, G. W. Trucks, H. B. Schlegel, G. E. Scuseria, M. A. Robb, J. R. Cheeseman, G. Scalmani, V. Barone, G. A. Petersson, H. Nakatsuji, X. Li, M. Caricato, A. V. Marenich, J. Bloino, B. G. Janesko, R. Gomperts, B. Mennucci, H. P. Hratchian, J. V. Ortiz, A. F. Izmaylov, J. L. Sonnenberg, D. Williams-Young, F. Ding, F. Lipparini, F. Egidi, J. Goings, B. Peng, A. Petrone, T. Henderson, D. Rana-singhe, V. G. Zakrzewski, J. Gao, N. Rega, G. Zheng, W. Liang, M. Hada, M. Ehara, K. Toyota, R. Fukuda, J. Ha-segawa, M. Ishida, T. Nakajima, Y. Honda, O. Kitao, H. Nakai, T. Vreven, K. Throssell, J. A. Montgomery, Jr., J. E. Peralta, F. Ogliaro, M. J. Bearpark, J. J. Heyd, E. N. Brothers, K. N. Kudin, V. N. Staroverov, T. A. Keith, R. Kobayashi, J. Normand, K. Raghavachari, A. P. Rendell, J. C. Burant, S. S. Iyengar, J. Tomasi, M. Cossi, J. M. Millam, M. Klene, C. Adamo, R. Cammi, J. W. Ochterski, R. L. Martin, K. Morokuma, O. Farkas, J. B. Foresman, and D. J. Fox, Gaussian, Inc., Wallingford CT, 2019.
